# Impact of amyloid β aggregate maturation on antibody treatment in APP23 mice

**DOI:** 10.1186/s40478-015-0217-z

**Published:** 2015-07-04

**Authors:** Karthikeyan Balakrishnan, Ajeet Rijal Upadhaya, Julia Steinmetz, Julia Reichwald, Dorothee Abramowski, Marcus Fändrich, Sathish Kumar, Haruyasu Yamaguchi, Jochen Walter, Matthias Staufenbiel, Dietmar Rudolf Thal

**Affiliations:** Institute of Pathology – Laboratory of Neuropathology, Center of Biomedical Research, University of Ulm, Helmholtzstrasse 8/1, D-89081 Ulm, Germany; Novartis Pharma, Novartis Institutes for Biomedical Research, Basel, Switzerland; Institute for Pharmaceutical Biotechnology, Center of Biomedical Research, University of Ulm, Ulm, Germany; Department of Neurology, University of Bonn, Bonn, Germany; Gunma University School of Health Sciences, Maebashi, Gunma Japan; Department of Cellular Neurology, Hertie Institute for Clinical Brain Research, University of Tübingen and DZNE, German Center for Neurodegenerative Diseases, Tübingen, Germany

**Keywords:** Amyloid, Immunization, Antibody, Protofibrils, Fibrils, Clearance

## Abstract

**Introduction:**

The deposition of the amyloid β protein (Aβ) in the brain is a hallmark of Alzheimer's disease (AD). Removal of Aβ by Aβ-antibody treatment has been developed as a potential treatment strategy against AD. First clinical trials showed neither a stop nor a reduction of disease progression. Recently, we have shown that the formation of soluble and insoluble Aβ aggregates in the human brain follows a hierarchical sequence of three biochemical maturation stages (B-Aβ stages). To test the impact of the B-Aβ stage on Aβ immunotherapy, we treated transgenic mice expressing human amyloid precursor protein (APP) carrying the Swedish mutation (KM670/671NL; APP23) with the Aβ-antibody β1 or phosphate-buffered saline (PBS) beginning 1) at 3 months, before the onset of dendrite degeneration and plaque deposition, and 2) at 7 months, after the start of Aβ plaque deposition and dendrite degeneration.

**Results:**

At 5 months of age, first Aβ aggregates in APP23 brain consisted of non-modified Aβ (representing B-Aβ stage 1) whereas mature Aβ-aggregates containing N-terminal truncated, pyroglutamate-modified Aβ_N3pE_ and phosphorylated Aβ (representing B-Aβ stage 3) were found at 11 months of age in both β1- and PBS-treated animals. Protective effects on commissural neurons with highly ramified dendritic trees were observed only in 3-month-old β1-treated animals sacrificed at 5 months. When treatment started at 7 months of age, no differences in the numbers of healthy commissural neurons were observed between β1- and PBS-treated APP23 mice sacrificed with 11 months.

**Conclusions:**

Aβ antibody treatment was capable of protecting neurons from dendritic degeneration as long as Aβ aggregation was absent or represented B-Aβ stage 1 but had no protective or curative effect in later stages with mature Aβ aggregates (B-Aβ stage 3). These data indicate that the maturation stage of Aβ aggregates has impact on potential treatment effects in APP23 mice.

**Electronic supplementary material:**

The online version of this article (doi:10.1186/s40478-015-0217-z) contains supplementary material, which is available to authorized users.

## Introduction

The deposition of the amyloid β-protein (Aβ) in senile plaques is one of the hallmarks of Alzheimer’s disease (AD) [1, 2]. Active and passive immunization against the Aβ peptide has been developed to treat AD [3, 4]. In amyloid precursor protein (APP) transgenic mice, peripheral administration of Aβ-antibodies lead to a reduced number of detectable plaques and prevented a further increase in the number of detectable Aβ plaques [3, 5]. Even the amount of fibrillar, Thioflavin S-positive plaques was reduced in anti-Aβ treated animals [6]. However, another group could not confirm Aβ reduction though an improvement of memory dysfunction was observed [7–9]. Active immunization in PDAPP mice demonstrated more effective rescue from cognitive impairment when treatment was started to prevent Aβ pathology compared to mice treated at a later point in life in a reversal trial [10]. First trials of active and passive Aβ immunization in AD patients, however, did not show a reduction of symptoms or prevention from further disease progression [11–16].

Given the discrepancy between the reduction of stained plaques and memory improvement in APP transgenic mice and the clinical disease progression in AD patients the question arises whether Aβ immunization failed in AD patients because treatment was started too late after the onset of morphologically detectable neurodegeneration. Recently, we reported biochemical stages of Aβ aggregate maturation (B-Aβ stages) in the course of preclinical and symptomatic AD [17]. In addition to the most abundant forms of Aβ, i.e. Aβ_1–40_ and Aβ_1–42,_ posttranslational modifications of Aβ have been identified in Aβ aggregates, including N-terminal truncations and pyroglutamate modifications at residues 3 or 11 (Aβ_N3pE_ and Aβ_N11pE_) and phosphorylation at Serine residue 8 (pAβ) [18–20]. The presence of Aβ_N3pE_, thereby, indicates B-Aβ stage 2 and that of pAβ B-Aβ stage 3 [17]. It is not yet clear whether the B-Aβ stage has impact on the effect of Aβ-antibody treatment and whether maturation of Aβ aggregates explains the failure of treatment trials in AD patients.

To address these questions in a mouse model we chose APP23 mice, which overexpress human APP with the Swedish mutation (KM670/671NL) driven by a Thy1 promoter [21]. These mice produce soluble and insoluble Aβ aggregates, Aβ plaques and they show neuron loss in CA1, loss of asymmetric synapses in the frontocentral neocortex [22, 23] as well as more subtle degeneration of a subtype of layer III commissural neurons in the frontocentral neocortex, i.e. the degeneration of commissural neurons with a highly ramified dendritic tree. Degeneration of these highly ramified commissural neurons was seen as early as at 5 months of age in parallel with the onset of Aβ plaque deposition whereas no such changes were reported at 3 months of age in the absence of Aβ aggregates [24]. Degeneration of axons of commissural neurons represents the morphological correlative for corpus callosum atrophy, which is an early event in the pathogenesis of AD [25–27]. As such, these mice showing early degeneration of commissural neurons are ideally suited to study the effects of Aβ-antibody treatment on these Aβ-related neurodegenerative changes.

In this study, we treated APP23 mice with the Aβ-antibody β1 (Additional file 1: Table S1) beginning 1) at 3 months of age before the onset of plaque deposition and degeneration of commissural neurons and 2) at 7 months of age after plaque deposition and neurodegeneration started. The β1 antibody is directed against the N-terminus of Aβ and capable of preventing Aβ deposition in APP23 mice when it is transgenetically expressed [28]. The treated animals were sacrificed and the samples were analyzed 1) with 5 months - when first plaques and early dendrite degeneration can be identified in APP 23 mice - to detect protective effects and 2) with 11 months to clarify whether β1 antibody treatment allows recovery of altered highly ramified commissural neurons after a 4-month-treatment period. PBS-treated animals were used as controls.

## Material and methods

### Animals

APP23 mice were generated as described previously [21] and continuously back-crossed to C57BL/6. The murine Thy-1 cassette was used to drive neuron-specific expression of human APP751 with the Swedish double mutation 670/671 KM → NL. Heterozygous female APP23 mice of two age groups, 3 months (*n* = 44) and 7 months, (*n* = 39) were treated in this study and analyzed at 5 and 11 months of age, respectively. Animals were treated in agreement with German and Swiss laws on the use of laboratory animals.

### Vaccination

Passive vaccination was performed in APP23 mice by weekly intraperitoneal (i.p.) injections of 500 μg of β1-anti-Aβ antibody [29] (Additional file 1: Table S1). As control group, APP23 mice received weekly i.p. injections of phosphate-buffered saline (PBS).

3-month-old animals were treated for 9 weeks, until 5 months of age, and were sacrificed 2–3 days after the last injection (β1: *n* = 21, PBS: *n* = 23). 7-month-old mice received injections for 12 weeks, until the age of 11 months, and were sacrificed for analysis 5–6 days after the last injection (β1: *n* = 21, PBS: *n* = 18). Animal experiments were carried out with permission of the Regierungspräsidium Tübingen/Germany (Permission: Animal Experiment No. 933) and the Animal Care and Use Committees of the Kanton Basel, Switzerland (Permission 1980).

### Tissue preparation and DiI tracing

For DiI tracing and histopathological examinations, the brains of 5 and 11-months-old β1-treated and PBS-treated animals were used (5 months – β1: *n* = 9, PBS: *n* = 10; 11 months – β1: *n* = 10; PBS: *n* = 10). Mice were anesthetized and perfusion was performed transcardially with Tris-buffered saline (TBS) with heparin (pH 7.4) followed by the injection of 0.1 M PBS (pH 7.4) containing 2.6 % paraformaldehyde (PFA), 0.8 % iodoacetic acid, 0.8 % sodium periodate and 0.1 M D-L lysine. The brains were removed in total and post-fixed in 2.6 % phosphate-buffered PFA (pH 7.4) containing 0.8 % iodoacetic acid, 0.8 % sodium periodate and 0.1 M D-L lysine [30]. After 3 days, a single crystal (0.3 mm^3^) of the carbocyanine dye DiI (Molecular Probes, Eugene, OR, USA) was implanted into the left frontocentral cortex, 1 mm rostrally from the central sulcus, 2 mm laterally from the middle line and 1 mm deep in the cortex as reported earlier [24]. This dye allows precise Golgi-like tracing of neurons in post-mortem fixed tissue in a quality similar to *in-vivo* tracing methods [24]. After incubation in 2.6 % phosphate-buffered PFA for at least 3 months at 37 °C, 100 μm thick coronal vibratome sections were cut. All sections of a given mouse brain were separately stored and continuously numbered. Sections were temporarily mounted in TBS for microscopic analysis.

### Microscopic and quantitative analysis

In layer III of the frontocentral cortex of the right hemisphere, contralateral to the implantation site of the tracer, the morphology of traced commissural neurons was examined. The traced neurons were assigned to different types according to their morphology [24] (Additional file 2: Table S2). Then the number of traced commissural neurons of each type in β1-treated APP23 mice was counted and compared with that in PBS-treated APP23 mice. For qualitative and quantitative analysis, 10 consecutive sections (100-μm thickness each) representing a tissue block of 1 mm thickness were studied for each mouse. Analysis started at the anterior commissure setting the caudal limit of the investigated tissue block. For each coronal section, the medial boundary of the region investigated was set as the vertical line at the cingulum that separated the cingulate cortex from secondary motor cortex (M2). The horizontal boundary was set as the horizontal line separating the primary somatosensory cortex (S1) from the insular cortex.

For the qualitative analysis, a laser scanning confocal microscope (Leica TCS NT, Leica, Bensheim, Germany) was used. Stacks of 2D images were superimposed digitally using the ImageJ image processing and analysis software (National Institutes of Health (NIH), Bethesda, MD, USA), and 3D data sets were generated for the visualization of neurons with their entire dendritic tree. For quantification, traced neurons in layer III were counted in the region of interest in 10 consecutive sections of the tissue block taken for qualitative and quantitative analysis using a fluorescence microscope (Leica DMLB, Leica, Germany). In so doing, we analyzed a cortex volume of 5–6 mm^3^ in each mouse. Mean and median values of the number of traced neurons were calculated and compared between β1-treated and PBS-treated APP23 mice of a given age.

### Immunohistochemistry

Morphological and immunohistochemical analysis were carried out on sections of the traced animals after obtaining the tracing results. Vibratome sections of the frontocentral cortex were immunostained with anti-Aβ_17–24_, anti-Aβ_1–17_, anti-Aβ_42_, anti-Aβ_40_, anti-Aβ_N3pE_, anti-pAβ, anti-Aβ-β1, anti-APP, anti-mouse-IgG, anti-glial fibrillary acidic protein (GFAP) and the microglia marker iba-1 [18, 19, 29, 31, 32] (Additional file 1: Table S1). The primary antibodies were detected with the respective biotinylated anti-mouse and anti-rabbit IgG secondary antibodies and visualized with the ABC-complex (ABC-Kit, Vector Laboratories, Burlingame, CA, USA) and diaminobencidine-HCl (DAB) as chromogen. To avoid crossreactivity of intrinsic IgG with anti-mouse-IgG secondary antibodies, sections were preincubated with goat-anti-mouse-IgG [33]. Protofibrils and fibrils were detected with B10AP-antibody fragments coupled with alkaline phosphatase [34]. They were visualized with permanent red (DAKO, Glostrup, Denmark). For immunofluorescence, rabbit primary antibodies were detected with carbocyanine 3 (Cy3)-labeled anti-rabbit-IgG secondary antibodies whereas mouse IgG was detected with Cy2-labeled anti-mouse IgG antibodies without previous anti-mouse-IgG blocking to allow the detection of intrinsic IgGs. Amyloid material was identified in the double stained section by UV-light-induced amyloid autofluorescence, i.e. detection of unstained amyloid material by fluorescence microscopy (excitation filter: 360–370 nm; emission filter: > 420 nm) [35]. Respective positive and negative controls were performed.

### Quantification of plaque loads for Aβ_42_-, Aβ_40_-, Aβ_N3pE_-, pAβ-, β1- and B10AP-positive plaques

Aβ_42_ load was determined as the percentage of the area in the frontocentral cortex covered by Aβ plaques detected with anti-Aβ_42_ antibodies. Morphometry for Aβ_42_ load determination was performed using ImageJ image processing and analysis software by interactive measurement of plaque areas in a region of interest as well as of the total area of interest. The plaque load was calculated as the percentage of the area of interest covered by amyloid plaques stained with the antibody (National Institutes of Health, Bethesda, USA) [23]. Accordingly, the Aβ_40_, Aβ_N3pE_, pAβ, Aβ_1–17_, β1 and B10AP loads were determined as the percentage of the frontocentral cortex area covered by plaques positive for the respective antibodies.

### Protein extraction from brain tissue and serum

For biochemistry, deeply anesthetized mice were sacrificed by decapitation. Blood was collected after decapitation. Serum was isolated after centrifugation at 3000 × g for 30 min at room temperature. Serum samples were immediately frozen at −80 °C until the performance of the experiments. The brains were taken, dissected, and unfixed left and right hemispheres as well as the brain stem and the cerebellum were kept separately at −80 °C for further analysis.

Protein extraction from frozen brain samples of 5- and 11-month-old APP23 treated with β1-antibodies (5 months: *n* = 12, 11 months: *n* = 11) or PBS (5 months: *n* = 13, 11 months: *n* = 8) was carried out for biochemical studies [17, 23]. Briefly, fresh frozen forebrain tissue samples (0.4 g) were homogenized in 2 ml of 0.32 M sucrose dissolved in Tris-buffered saline (TBS) containing a protease and phosphatase inhibitor-cocktail (Complete and PhosSTOP, Roche, Mannheim, Germany) with Micropestle (Eppendorf, Hamburg, Germany) followed by sonication. The homogenate was centrifuged for 30 min at 14,000 × g at 4 °C. The supernatant (S1), containing both the soluble and dispersible fraction was kept for further ultracentrifugation. The pellet (P1) containing the membrane-associated and the insoluble, plaque-associated fraction was resuspended in 2 % SDS. Ultracentrifuging of the supernatant S1 at 175,000 × g was used to separate the soluble, i.e. the supernatant after ultracentrifuging (S2), from the dispersible fraction, i.e. the resulting pellet (P2). The pellet P2 with the dispersible fraction was resuspended in TBS.

The SDS-resuspended pellet P1 was centrifuged at 14,000 × g the supernatant (S3) was kept as membrane-associated SDS-soluble fraction. The pellet (P3) that remained was dissolved in 70 % formic acid and dried in a vacuum centrifuge (Vacufuge, Eppendorf, Hamburg, Germany) and reconstituted in 100 μl of 2X LDS (lithium dodecyl sulfate) sample buffer (Life Technologies, Carlsbad, CA, USA) followed by heating at 70 °C for 5 min. The resultant sample was considered as insoluble, plaque-associated fraction [36]. The total protein amounts of soluble, dispersible, and membrane-associated fractions were determined using BCA Protein Assay (Bio-Rad, Hercules, CA, USA).

### Immunoprecipitation

For immunoprecipitation (IP), 200 μl of the native soluble and dispersible fractions from the brain lysates were incubated with 1 μl A11 antibodies against non-fibrillar oligomers, or 5 μl B10AP antibody fragments for precipitation of protofibrils and fibrils (Additional file 1: Table S1) or were kept without adding antibodies to identify already antibody-bound Aβ. 50 μl of protein G-coated Microbeads (Miltenyi Biotec, Bergisch-Gladbach, Germany) were added to the mixture and incubated overnight at 4 °C. The mixture was then passed through the μColumns, which separate the microbeads by retaining them in the column, while the rest of the lysate flows through. After one mild washing step with 1X TBS at pH 7.4 the microbead-bound-proteins were eluted with 95 °C heated 1X LDS sample buffer (Life Technologies, Carlsbad, CA, USA).

### SDS-PAGE and Western blot analysis

For SDS-PAGE, soluble (S2), dispersible (P2), membrane-associated (SDS-soluble; S3), insoluble, plaque-associated (formic acid soluble; P3) fractions and IP eluates (50 μg total protein) were electrophoretically resolved in a precast NuPAGE 4–12 % Bis-Tris gel system (Life Technologies, Carlsbad, CA, USA). Proteins were transferred onto Nitrocellulose membrane and membranes were boiled in 1X PBS for 5 min. The protein load was controlled either by Ponceau S staining (C4, 1/1000, Santa Cruz Biotechnology, Santa Cruz, CA, USA) or by MemCode reversible protein stain kit (Pierce, Rockford, IL, USA) prior to immunoblotting.

Aβ was detected by western blotting with anti-Aβ_1–17_, anti-pAβ and anti-Aβ_N3pE_ antibodies (Additional file 1: Table S1). Blots were incubated with chemiluminescent ECL detection system (Supersignal Pico Western system, ThermoScientific-Pierce, Waltham, MA, USA) or Luminata Forte Western HRP substrate (Merck Millipore, Billerica, Massachusetts, USA) and acquired using either ECL Hyperfilm (GE Healthcare, Buckinghamshire, UK) or CCD imager Image Quant LAS 4000 (GE Healthcare, Buckinghamshire, UK). Since anti-Aβ_1–17_ also stains APP we used the respective protein bands corresponding to ~110 kDa seen with this antibody as internal loading control to verify the protein content.

Because Aβ aggregates readily dissociate in the presence of SDS-containing buffers into monomers and small oligomers, such as dimers, trimers, or Aβ*56 [37, 38], we analyzed differences among the monomer bands that indicate changes in the protein levels of precipitated Aβ aggregates densitometrically using ImageJ software (NIH, Bethesda, MD, USA). This method allows a semiquantitative assessment of Aβ [23]. Briefly, the X-ray films were scanned and image colors were inverted or the images acquired by CCD imager were exported as 8-bit grayscale TIFF files. The relative protein levels of the monomer bands were measured as integrated density values for each lane [39].

Biochemical stages of Aβ aggregation (B-Aβ stages) were determined on the basis of Aβ, Aβ_N3pE_ and pAβ detection in the soluble, dispersible, membrane-associated, and plaque-associated fraction [17]: B-Aβ stage 1 = Aβ aggregates detected only with anti-Aβ_1–17_; B-Aβ stage 2 = Aβ aggregates positive for anti-Aβ_1–17_ and anti-Aβ_N3pE_ but not for anti-pAβ; B-Aβ stage 3 = Aβ aggregates containing anti-Aβ_1–17_-, anti-Aβ_N3pE_- and anti-pAβ-positive material.

### Stereology

Six β1-treated and six PBS-treated APP23 mice at the ages of 5 and 11 months, respectively, were chosen randomly for stereology. One brain section including the hippocampal formation already quantified for the number of DiI-traced neurons was selected by chance and stained with aldehyde fuchsin-Darrow red. Quantification of neurons was performed according to the principles of unbiased stereology [40]. The CA1 volume was measured in serial 100 μm thick sections of the entire mouse brain at 5 × magnification. Neurons were counted in three different, randomly chosen microscopic fields (40 × objective magnification) of an aldehyde fuchsin – Darrow red stained section of the frontocentral cortex and CA1, respectively. For optical dissection, stacks of 10 images in 2 μm focus distance were generated for each microscopic field. Only those neurons having nuclei with dark and round nucleoli visible in the center of soma in one of the stack-images were considered for quantification using the ImageJ software (NIH, Bethesda, USA). The number of neurons in the frontocentral cortex and CA1 was calculated on the basis of the respective reference volumes and neuron densities as previously published [41].

### Electron microscopy, immunoelectron microscopy and semiquantitative assessment of synapse densities

100 μm thick vibratome sections of the frontocentral cortex from six β1-treated and six PBS-treated APP23 mice, aged 5 and 11 months, respectively, were flat-embedded in Epon (Fluka, Germany). A part of the of the frontocentral cortex covering all six layers was cut and pasted on a second Epon block ultrathin sections were cut at 70 nm. Epon sections were block stained with uranyl acetate and lead citrate, and viewed with a Zeiss EM10 (Zeiss, Oberkochen, Germany), or a JEM-1400 (JEOL, Tokyo, JP) electron microscope. Digital pictures were taken.

Synaptic densities in the frontocentral cortex were measured in 20 randomly taken pictures of the layers II-VI at 4600-times magnification. The numbers of the symmetric and asymmetric synapses were counted and the length of the synapses was determined with the ImageJ software (NIH, Bethesda, USA). The synaptic density was determined separately for symmetric and asymmetric synapses according to DeFelipe et al. [42] (synaptic density = number of synapse-profiles in a given area / length of synaptic profiles). These semiquantitative data were used to compare the synaptic densities between the different mouse lines. Asymmetric and symmetric synapses were distinguished according to published criteria [43, 44].

### Statistical analysis

SPSS 21.0 (SPSS, Chicago, IL, USA) software was used to calculate statistical tests. Non-parametric tests were used to compare β1-treated and PBS-treated APP23 mice. *p*-values were corrected for multiple testing using the Bonferroni-method. Parametric data were analyzed by ANOVA with subsequent Games-Howell post-hoc test to correct for multiple testing or by using the Welch test. The results of the statistical analysis are summarized in Additional file 3: Table S3.

## Results

### Neurodegeneration in β1-treated and PBS-treated APP23 mice

**Fig. 1:**
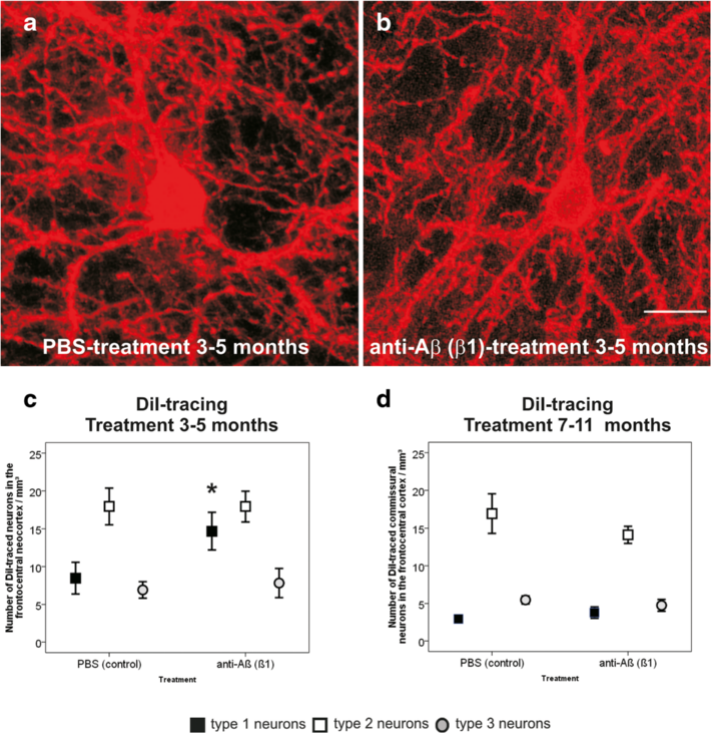
Degeneration of commissural neurons with a highly ramified dendritic tree: Effects of β1 antibody treatment. **a**, **b** Layer III type 1 commissural neurons in the frontocentral cortex traced with DiI. The detectable type 1 commissural neurons did not show significant differences between PBS- and β1-treated APP23 mice even at 5 months of age. **c** The number of the type 1 commissural neurons in 5-month-old β1 treated mice was higher than that in PBS-treated animals whereas no such differences were observed for type 2 and 3 commissural neurons. **d** No differences in the numbers of DiI-traced type 1–3 commissural neurons were seen among 11-month-old PBS- and β1-treated APP23 mice. Graphs represent mean values (symbols) and standard errors (whiskers), * *p* < 0.05 (Statistical analysis in Additional file 3: Table S3). Calibration bar in **b** (valid for **a**, **b**) = 37.5 μm

Between 3 and 5 months of age degeneration of dendrites of DiI-traced highly ramified commissural layer III neurons in the frontocentral cortex was observed in β1-treated as well as in PBS-treated animals (Fig. 1). However, β1-treated mice showed slightly higher numbers of type I commissural neurons than PBS-treated animals (Mann-Whitney *U*-test: *p* < 0.05). The numbers of type II and III commissural neurons did not differ between β1- and PBS-treated animals (Fig. 1 and Additional file 3: Table S3). Treatment with the β1-antibody between 7 and 11 months of age had no obvious effect on the numbers of type I, II and III layer III commissural neurons traced in the frontocentral neocortex in comparison to PBS-treated controls (Fig. 1 and Additional file 3: Table S3a).

Significant differences in the synapse densities of asymmetric and symmetric synapses in the frontocentral cortex as well as differences in the numbers of CA1 neurons were not observed between β1-treated and PBS-treated animals in both age groups (Additional file 3: Table S3b, c and Additional file 4: Figure S1).

### Aβ plaques in β1-treated and PBS-treated APP23 mice

**Fig. 2:**
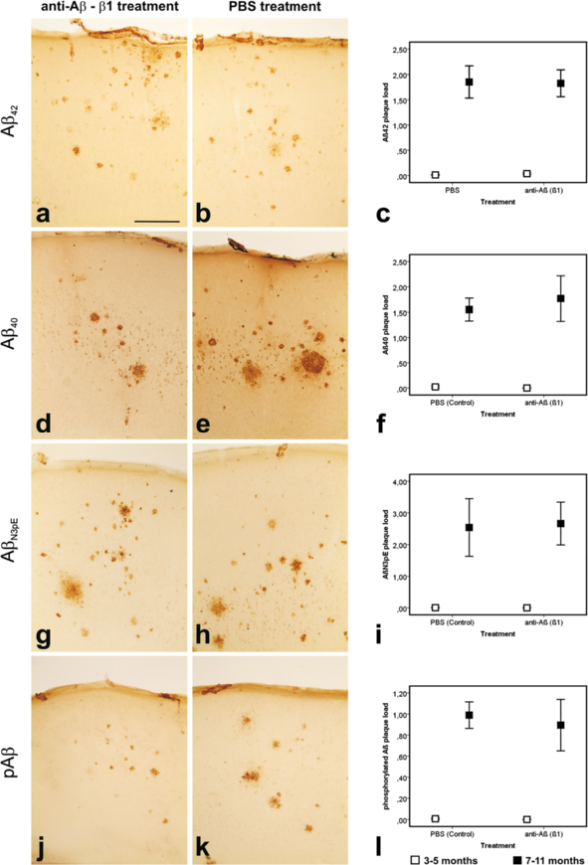
Aβ plaque pathology in the frontocentral cortex of APP23 mice: Effects of β1 antibody treatment. Aβ plaque pathology in PBS- and β1-treated APP23 mice. The staining pattern in 11-month-old mice as well as the plaque loads at 5 and 11 months of age did not differ between PBS- and anti-Aβ (β1) treated animals when staining the plaques with antibodies raised against Aβ_42_ (**a**–**c**), Aβ_40_ (**d**–**f**), Aβ_N3pE_ (**g**–**i**), and pAβ (**j**–**l**). Graphs **c**, **f**, **i**, and **l** represent mean values (symbols) and standard errors (whiskers). (Statistical analysis see Additional file 3: Table S3). Calibration bar in **a** (valid for **a**, **b**, **d**, **e**, **g**, **h**, **j**, **k**) = 280 μm

**Fig. 3:**
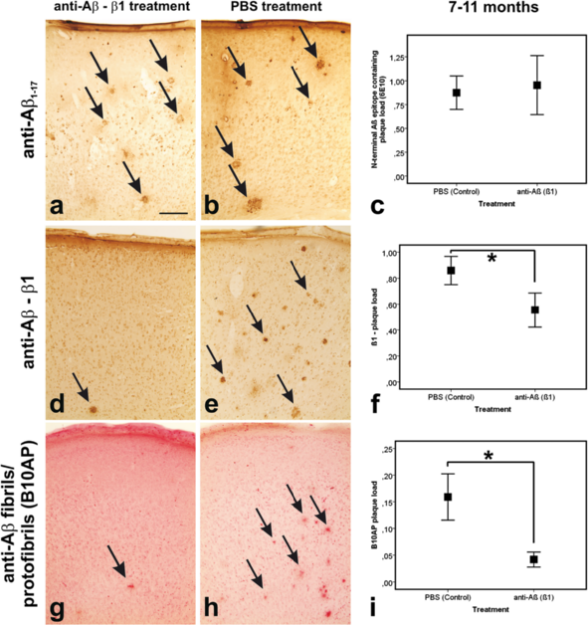
Epitope masking effects of β1 antibody treatment in the frontocentral cortex of APP23 mice. Epitope modification after anti-Aβ (β1) antibody treatment in 11-month-old APP23 mice. Although an antibody raised against Aβ_1–17_ (6E10) exhibited similar levels of Aβ plaques (**a**, **b** - arrows) as provided by the respective Aβ (6E10) plaque load (**c**) the β1 antibody used for treatment detected less plaques in the treated animals than in the PBS-controls (**d**, **e** - arrows) as confirmed by a significantly lowered β1 plaque load (**f**). Less plaques were also detected with B10AP antibody fragments detecting protofibril/ fibril-specific epitopes (**g**, **h** - arrows) with respective changes in the B10AP plaque load (**i**). Graphs **c**, **f**, and **i** represent mean values (symbols) and standard errors (whiskers), * *p* < 0.05 (Statistical analysis in Additional file 3: Table S3). Calibration bar in **a** (valid for **a**, **b**, **d**, **e**, **g**, **h**) = 280 μm

Aβ plaques detectable with antibodies directed against Aβ_42_, Aβ_40_, Aβ_17–24_, Aβ_1–17_, Aβ_N3pE_, and pAβ were found in both, β1-treated and PBS-treated APP23 mice (Figs. 2 and 3), without quantitative differences at 5 and 11 months of age, respectively (Additional file 3: Table S3d). At 5 months of age only single plaques in some of the mice were observed whereas in 11-month-old APP23 mice moderate numbers of plaques were seen. Further analysis of 11-month-old animals using the β1 antibody (used for passive immunization) showed that treated animals exhibited fewer plaques stained with β1 indicating epitope masking (Fig. 3 and Additional file 3: Table S3d). B10AP-positive plaques were also reduced in β1-treated 11-month-old animals (Fig. 3) whereas 5-month-old mice did not exhibit B10AP-positive plaques regardless of β1 treatment (Additional file 3: Table S3d). APP-positive dystrophic neurites were seen in APP-type neuritic plaques (i.e. amyloid plaques associated with APP-positive dystrophic neurites) of β1-treated and PBS-treated APP23 mice (Additional file 5: Figure S2). Mouse IgG was not observed in plaques of 5-month-old animals whereas amyloid plaques in 11-month-old β1- and PBS-treated mice exhibited IgG in similar amounts (Additional file 6: Figure S3 and Additional file 7: Figure S4). Plaque-associated astrocytes and microglial cells were seen in both β1- and PBS-treated APP23 mice at 11 months of age whereas no glial reaction was evident in 5-month-old animals (Additional file 6: Figure S3 and Additional file 7: Figure S4).

### Brain Aβ, Aβ_N3pE_, and pAβ in β1-treated and PBS-treated APP23 mice

**Fig. 4:**
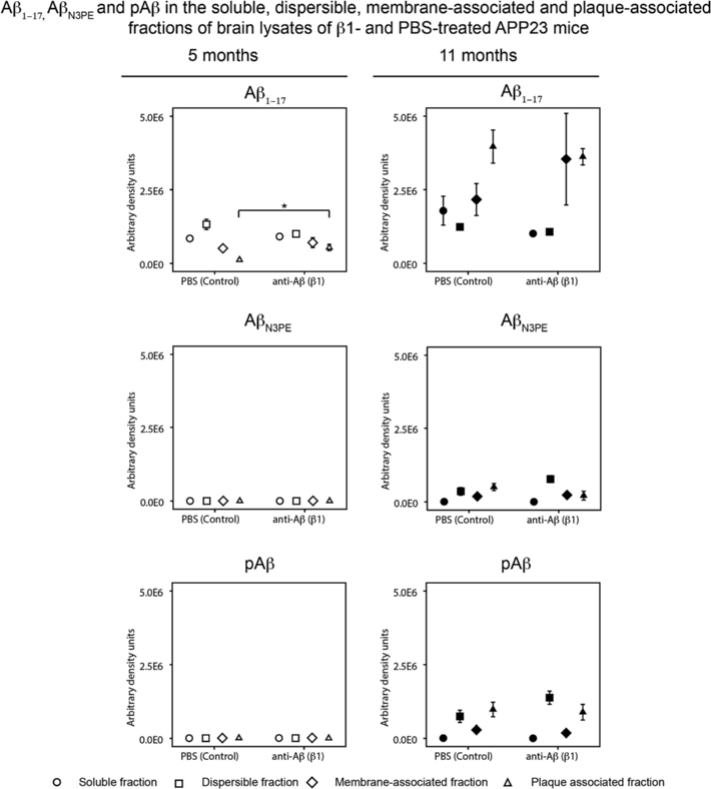
Soluble, dispersible, membrane-associated, and plaque-associated Aβ in APP23 mice: Effects of β1 antibody treatment. Semiquantitative comparison of Aβ, Aβ_N3pE_, and pAβ in the soluble, dispersible, membrane-associated, and plaque-associated fraction of brain homogenates of PBS- and β1-treated 5- and 11-month-old APP23 mice received by quantification of western blots displayed in Additional file 8: Figure S5. No differences between PBS- and β1-treated animals except for plaque-associated Aβ in 5-month-old mice: β1-treated animals exhibited slightly more non-modified plaque-associated Aβ than non-treated mice. Aβ_N3pE_ and pAβ were not detected in brain homogenates of 5-month-old APP23 mice but in the dispersible, membrane-associated, and plaque-associated fraction of 11-month-old mice without differences in relation to the treatment. Graphs represent mean values (white symbols 5-month-old mice; black symbols 11-month-old mice) and standard errors (whiskers). (* *p* < 0.05; detailed Statistical analysis in Additional file 3: Table S3)

Western blot analysis of forebrain homogenates revealed no obvious differences in the amounts of soluble, dispersible, and membrane-associated Aβ detectable with anti-Aβ_1–17_ in β1-treated and PBS-treated animals of both age groups (at 5 and 11 months of age). β1-treated 5-month-old APP23 mice exhibited higher amounts of anti-Aβ_1–17_ detected plaque-associated Aβ than PBS-treated animals (Fig. 4, Additional file 3: Table S3e and Additional file 8: Figure S5). In 11-month-old mice, Aβ in all four fractions was elevated in comparison to 5-month-old APP23 mice. Differences between the treatment groups were not obvious at this age (Fig. 4, Additional file 3: Table S3e and Additional file 8: Figure S5). Aβ_N3pE_ and pAβ were not observed in brain homogenates of 5-month-old animals but in mice of 11 months of age. No differences in the pattern of Aβ_N3pE_ and pAβ aggregation between β1- and PBS-treated animals were seen (Fig. 4, Additional file 3: Table S3e and Additional file 8: Figure S5). In accordance with a previously published hierarchical pattern of Aβ aggregate maturation during the course of AD, the presence of Aβ in the absence of biochemically detectable Aβ_N3pE_ and pAβ in 5-month-old APP23 mice corresponded to B-Aβ stage 1 whereas Aβ aggregates in 11-month-old mice consisting of normal Aβ, Aβ_N3pE_, and pAβ were referred to as B-Aβ stage 3 [17].

**Fig. 5:**
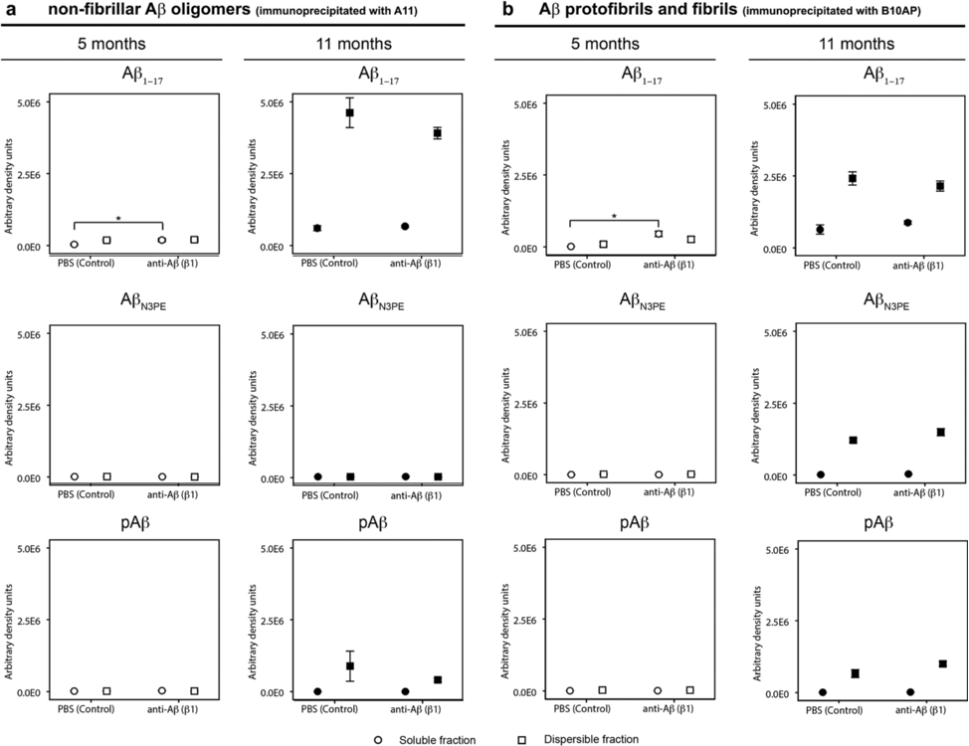
Soluble and dispersible (insoluble) Aβ fibrils/protofibrils and non-fibrillar oligomers in APP23 mice: Effects of β1 antibody treatment. Semiquantitative analysis of western blots from soluble and dispersible non-fibrillar oligomers immunoprecipitated with A11 and protofibrils and fibrils immunoprecipitated with B10AP from the respective brain homogenate fractions in 5- and 11-month-old β1- and PBS-treated APP23 mice. 5-month-old β1-treated mice exhibited soluble Aβ-oligomers, protofibrils and fibrils that were not seen in PBS-treated controls (* *p* < 0.05; detailed statistical analysis in Additional file 3: Table S3). Although dispersible Aβ oligomers, protofibrils and fibrils occurred in 5-month-old APP23 mice as well there were no differences between β1- and PBS-treated mice. Aβ_N3pE_ and pAβ were not seen in 5-month-old mice. No differences in soluble and dispersible Aβ aggregates were observed between 11-month-old β1- and PBS-treated mice. Aβ_N3pE_ and pAβ were not detectable in soluble oligomers, protofibrils and fibrils as well as in dispersible oligomers at both ages whereas dispersible fibrils and protofibrils exhibited both Aβ_N3pE_ and pAβ. Graphs represent mean values (white symbols 5-month-old mice; black symbols 11-month-old mice) and standard errors (whiskers). (Full blots Additional file 9: Figure S6; Statistical analysis Additional file 3: Table S3)

In 5-month-old mice, immunoprecipitation with the oligomer-specific A11 antibody and with protofibril- and fibril-specific B10AP antibody fragments and subsequent western blot analysis with anti-Aβ_1–17_ showed more Aβ oligomers, protofibrils and fibrils in the β1-treated group compared to the PBS-treated group selectively in the soluble fraction (Fig. 5, Additional file 3: Table S3 and Additional file 9: Figure S6). Such a difference was not found in 11-month-old animals. In the dispersible fractions there were no differences in the amounts of Aβ oligomers, protofibrils and fibrils between the β1-treated and PBS-treated animals of both age groups (Fig. 5, Additional file 3: Table S3f and Additional file 9: Figure S6).

In 5-month-old APP23 mice, irrespective of the treatment, no Aβ_N3pE_ and no pAβ was detected, in the soluble and dispersible fraction of samples immunoprecipitated with A11 or B10AP. Similarly, in 11-month-old mice, Aβ_N3pE_ and pAβ were not detected in A11 and B10AP immunoprecipitated oligomers, protofibrils and fibrils from the soluble fraction. Aβ_N3pE_ and pAβ were found in the dispersible fraction in Aβ protofibrils and fibrils precipitated with B10AP at 11 months of age but without significant differences among β1- and PBS-treated animals (Fig. 5, Additional file 3: Table S3f and Additional file 9: Figure S6). There was no detectable Aβ_N3pE_ in both groups of 11-month-old mice in the dispersible fractions immunoprecipitated with non-fibrillar Aβ oligomer-specific A11 antibodies but pAβ was observed in β1- and PBS-treated mice (Fig. 5, Additional file 3: Table S3f and Additional file 9: Figure S6).

**Fig. 6:**
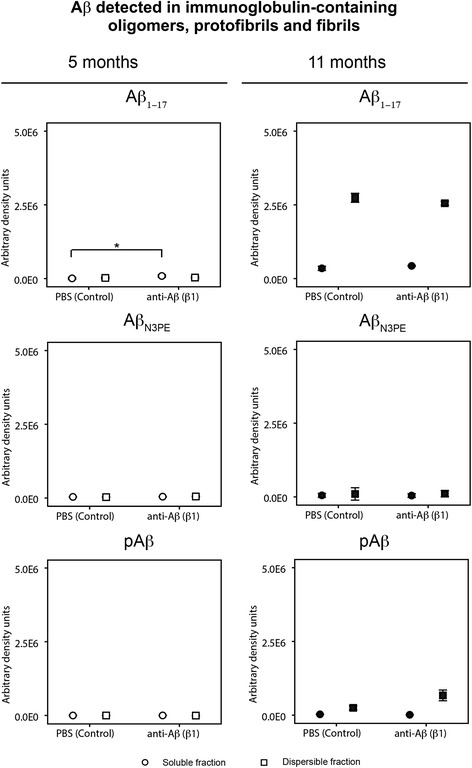
Immunoglobulin-bound Aβ detected in oligomers, protofibrils and fibrils in APP23 mice: Effects of β1 antibody treatment. Semiquantitative analysis of western blots after immunoprecipitation of immunoglobulin (antibody)-bound Aβ by precipitation of antibodies with protein G-coated magnetic beads. Subsequent western blot analysis with anti-Aβ_1–17_ revealed antibody-bound Aβ in the dispersible fraction of both β1- and PBS-treated mice at both ages. In 5-month-old β1-treated APP23 mice antibody-bound Aβ was found in the soluble fraction whereas no antibody-bound Aβ was precipitated in PBS-treated animals (* *p* < 0.05). At 11-months of age both, β1 and PBS-treated animals exhibited antibody bound soluble Aβ in similar amounts. Antibody-bound Aβ_N3pE_ was not observed whereas 11-month-old (but not 5-month-old) APP23 mice showed similar amounts of dispersible antibody-bound pAβ. No soluble antibody-bound pAβ was seen. Graphs represent mean values (white symbols 5-month-old mice; black symbols 11-month-old mice) and standard errors (whiskers). (Full blots Additional file 10: Figure S7; Statistical analysis in Additional file 3: Table S3)

Immunoprecipitation of antibody-bound Aβ by incubation of the brain samples with protein G magnetic beads without previous coupling to primary antibodies showed IgG-bound soluble Aβ in 5-month-old β1-treated mice detected with anti-Aβ_1–17_ antibodies, but not in PBS-treated animals (Fig. 6, Additional file 3: Table S3 and Additional file 10: Figure S7). Such a difference was not observed in 11-month-old mice. No soluble IgG-bound Aβ_N3pE_ and pAβ was seen in 5- and 11-month-old APP23 mice (Fig. 6). In the dispersible fractions, immunoprecipitation with protein-G coated magnetic beads without primary antibody exposure and subsequent western blotting with anti-Aβ_1–17_ antibodies, showed detectable amounts of IgG-bound Aβ in both age and treatment groups, respectively, without significant differences among treatment (Fig. 6, Additional file 3: Table S3g and Additional file 10: Figure S7). Aβ_N3pE_ was not seen in antibody-bound aggregates (Fig. 6, Additional file 3: Table S3g and Additional file 10: Figure S7). In 5-month-old APP23 mice no dispersible pAβ was observed in antibody-bound aggregates but 11-month-old animals exhibited dispersible pAβ in the precipitates without significant differences between the treatment groups (Fig. 6, Additional file 3: Table S3g and Additional file 10: Figure S7).

### Detection of Aβ in the blood serum of β1-treated and PBS-treated APP23 mice

**Fig. 7:**
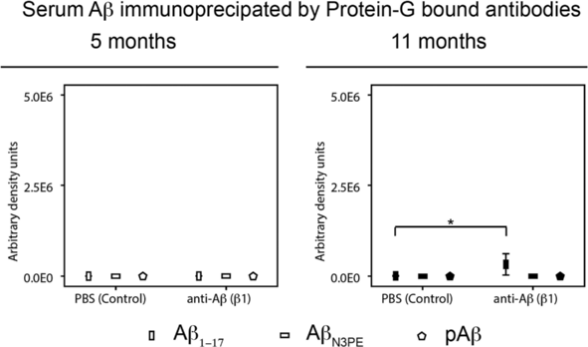
Serum Aβ immunoprecipitated by protein-G bound antibodies in APP23 mice: Effects of β1 antibody treatment. Semiquantitative assessments of western blot analysis of blood serum for Aβ and immunoprecipitation of intrinsic serum antibodies by incubation with protein G-coated magnetic beads and with subsequent western blotting for Aβ. Aβ was only seen in 5-month-old β1-treated APP23 mice after antibody-immunoprecipitation and detection with anti-Aβ_1–17_ (6E10) (* *p* < 0.05). Aβ_N3pE_ and pAβ were not found in these precipitates. PBS-treated and 5-month-old mice did not exhibit detectable amounts of Aβ. Graphs represent mean values (white symbols 5-month-old mice; black symbols 11-month-old mice) and standard errors (whiskers). (Full blots Additional file 11: Figure S8; Statistical analysis in Additional file 3: Table S3)

Western blot analysis did not reveal detectable amounts of Aβ in the serum of 5- and 11-month-old β1- and PBS-treated APP23 mice (Fig. 7 and Additional file 11: Figure S8). Only after immunoprecipitation of antibody-bound Aβ from blood serum using protein-G coated magnetic beads without previous primary antibody coupling showed anti-Aβ_1–17_ detectable Aβ in β1-treated 11-month-old APP23 mice but not in PBS-treated animals (Fig. 7, Additional file 3: Table S3 and Additional file 11: Figure S8). This effect was not observed at 5 months of age. Antibody-bound Aβ_N3pE_ and pAβ were not found in the blood serum of APP23 mice of both ages regardless of β1-treatment (Fig. 7 and Additional file 11: Figure S8).

## Discussion

**Fig. 8:**
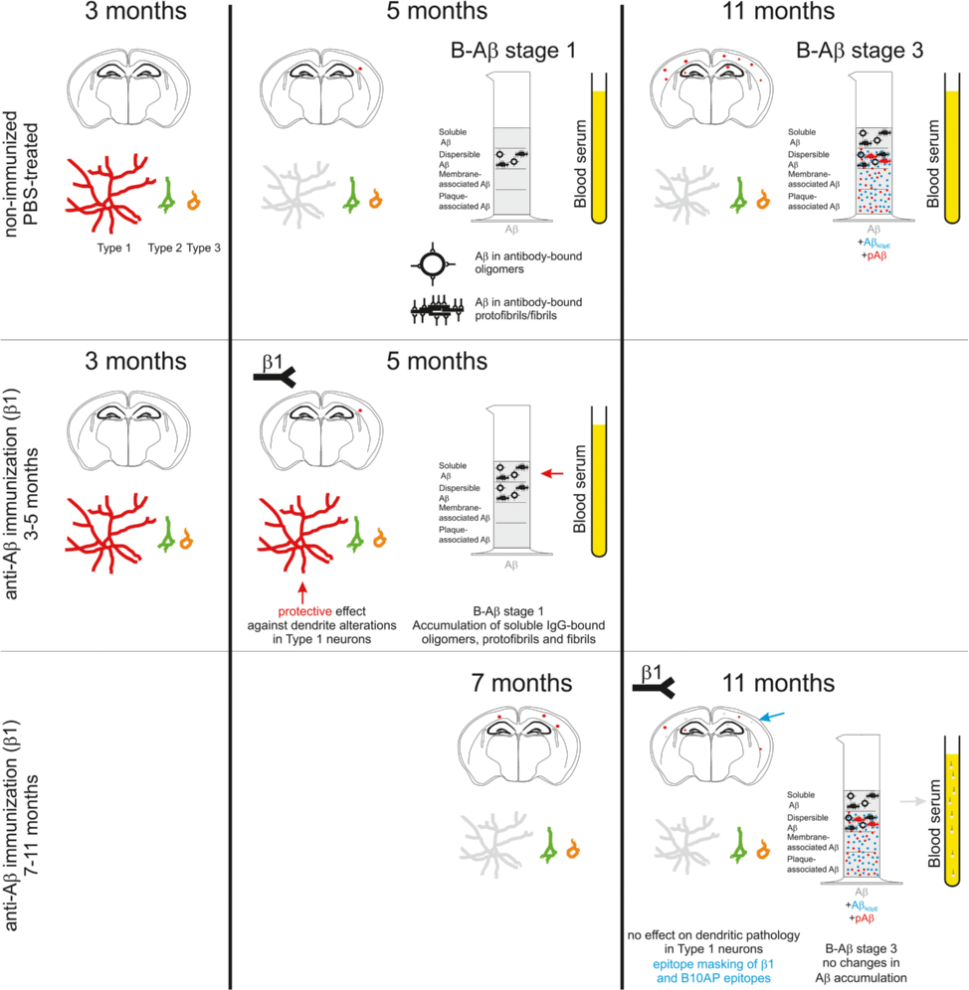
Effects of β1 antibody treatment in APP23 mice. Schematic representation of anti-Aβ (β1) treatment effects when applied before the onset of neurodegeneration and Aβ plaque deposition (3–5 months) and when provided with prevalent pathology (7–11 months). Anti-Aβ antibody treatment protected type 1 commissural neurons (Type 1) from dendritic degeneration accumulated antibody-bound oligomers, fibrils and protofibrils in the soluble fraction containing Aβ. These effects were seen in B-Aβ stage 1. No positive effect on Type 1 commissural neurons was found when β1-treatment was performed from 7 to 11 months of age. Moreover, plaque loads and biochemically detectable amounts of soluble, dispersible, membrane-associated, and plaque-associated Aβ were similar in β1- and PBS-treated APP23 mice. Aβ aggregation at 11 months of age corresponded to B-Aβ stage 3, i.e. fully mature, AD-related Aβ aggregates. Here, β1-treatment caused epitope modifications of Aβ aggregates resulting in less β1- and B10AP-positive plaques in treated mice. Moreover, at 11 months of age presence of antibody-bound non-modified Aβ in the serum was visible, whereas antibody-bound modified Aβ_N3pE_ and pAβ were not seen in the blood plasma

Our study on β1-immunized APP23 mice in comparison to non-immunized mice revealed five major findings (Fig. 8): 1. A qualitative change of Aβ aggregate composition was observed between 5- and 11-month-old APP23 mice corresponding with the biochemical maturation of Aβ aggregates as also seen in human AD brain. 2. Passive immunization with β1 antibodies starting at 3 months of age prior to the onset of Aβ deposition and dendritic degeneration until 5 months of age provided a protective effect against dendritic degeneration. Such an effect was not seen when immunization started later at 7 months of age when plaques and dendritic degeneration were already present. 3. Soluble antibody-bound oligomeric, protofibrillar and fibrillar Aβ-containing aggregates were found in β1-treated 5-month-old APP23 mice but not in PBS-treated mice. In older animals soluble antibody-bound Aβ was observed in similar amounts in both β1- and PBS-treated APP23 mice. 4. Aβ immunization in animals with preexisting Aβ pathology showed epitope masking effects in Aβ plaques but no biochemically detectable differences of Aβ in the brain between β1- and PBS-treated mice. 5. An increase of antibody-bound non-modified Aβ in the blood serum, but not of antibody-bound Aβ_N3pE_ and pAβ was detected in β1-treated APP23 mice.

Thus, it is tempting to speculate that Aβ-antibody treatment with the β1-antibody is most effective in early stages of Aβ aggregate maturation whereas late stage aggregates containing Aβ_N3pE_ and pAβ may preclude sufficient clearance. In the following paragraphs, we discuss the hypothesis that biochemical maturation of Aβ aggregates modifies its response to antibody treatment in detail.

### Protective effects of β1-antibody treatment in APP23 mice without preexisting pathology (Fig. 8)

Our study showed that β1 Aβ antibody treatment protected APP23 mice from severe dendrite degeneration in type 1 commissural neurons when treatment was started at 3 months before Aβ and dendrite pathology develop in this mouse model [24]. Such an effect was not observed when treatment was started later, i.e. after the onset of Aβ plaque pathology and type 1 commissural neuron degeneration with 7 months of age. This finding is in line with a previous report showing the absence of beneficial effects after γ-secretase treatment in mice with preexisting plaques [45]. The fact that only tracing of commissural neurons revealed differences between β1- and PBS-treated APP23 mice but neither CA1 neuron numbers nor synapse densities in the frontocentral cortex as further markers for neurodegeneration appear to be related to the APP23 mouse model. Different types of neurons differ with respect to their vulnerability to Aβ-related neurodegeneration in APP23 mice [24] and the type 1 commissural neurons are among the most vulnerable neurons in the frontocentral neocortex. Accordingly, degeneration of these neurons is a very sensitive indicator for Aβ-induced neurodegeneration in the APP23 mouse model that allows the detection of differences in Aβ-related neurodegeneration before other readouts such as synapse densities and neuron numbers become positive. It is important to note that the APP23 mouse is, in contrast to other APP-transgenic mice, the only APP-transgenic mouse that shows neuron loss, synapse loss, and dendritic degeneration [22, 24, 41]. Other mouse models showing such signs of neurodegeneration do not only express APP as the APP23 mouse but also presenilin 1 or τ that can also be responsible for neurodegeneration [46, 47]. As such our study in APP23 mice shows that morphological alterations of dendrites presumably caused by Aβ aggregates were subject of protection by immunotherapy but were not repaired once the neurons were fallen victim to degeneration.

Our finding of soluble antibody-bound Aβ-containing oligomers, protofibrils and fibrils as detected by immunoprecipitation in 5-month-old β1-treated APP23 mice that were not seen in PBS-treated animals indicates that β1-antibody treatment increases soluble Aβ aggregates. The antibody may stabilize existing soluble aggregates similar to blood Aβ [48, 49]. An enhancement of Aβ-aggregation cannot be excluded. The lack of degeneration suggests that such soluble Aβ-IgG aggregates are less pathogenic and may become subject of clearance. No antibody-bound Aβ aggregates were found in the blood of 5-month-old APP23 mice. The increase of antibody-bound Aβ in the blood of 11-month-old β1-treated APP23 mice but not in PBS-treated animals most likely reflects stabilization of plasma Aβ as previously described [48, 49]. No modified forms of Aβ were detected in the soluble fraction of 5- and 11-month old mice and in the blood due to their respective low concentrations or due to inefficient β1-antibody binding. β1-treatment did not cause a shift in soluble and insoluble Aβ aggregates in 11-month-old APP23 mice exhibiting Aβ_N3pE_ and pAβ. A possible explanation for the lack of a treatment effect on Aβ aggregates in 11-month-old APP23 mice may be the property of Aβ_N3pE_ and pAβ to stabilize Aβ aggregates *in-vitro* [18, 50] and, in so doing, to preclude sufficient antibody treatment effects as shown here.

Since the APP23 mouse is a mouse model that showed morphological signs of neurodegeneration with neuron loss in CA1 [22], reduction of asymmetric synapse densities [23], and dendritic degeneration in commissural neurons [24] this mouse model appears to be useful to study morphological signs of Aβ-related neurotoxicity to better understand the lack of improvement in anti-Aβ treated patients [11, 14, 16]. In this context, our results suggest that treatment attempts in already demented individuals exhibiting mature Aβ aggregates in the B-Aβ-stage 3 indicated by Aβ_N3pE_ and pAβ as its components [17] may not be successful because 1. irreversible degenerative changes already took place and 2. mature Aβ aggregates in B-Aβ stage 3 prevail [17], which were not removed by β1-antibody treatment in APP23 mice as described here. Accordingly, treatment strategies aimed at protecting from Aβ maturation and early Aβ-related neurodegenerative effects appear to be more attractive than those targeting non-modified forms of Aβ including oligomeric, protofibrillar and fibrillar aggregates.

Moreover, in human symptomatic AD cases in addition to Aβ plaque pathology, there are already high amounts of neurofibrillary tangles as well as severe neuron and synapse loss. As such, the amount of brain damage at this stage of the disease is significant. Given that most degenerating neurons are post-mitotic cells and will not be replaced [51] and that presence of Aβ plaques and neurofibrillary tangles induces gliosis [52–56] compensation or reconstruction of these lesions is very unlikely. Therefore, it is tempting to speculate that early B-Aβ stage preclinical AD cases are best suited to protect from or slow down progression of the disease as shown in 3-months-old β1-treated APP23 mice sacrificed at 5 months of age. Findings in APP23-β1-double transgenic mice clearly demonstrate that an efficient protective effect of the β1-antibody is possible in the initial stage of Aβ aggregation [28].

### Effects of β1-antibody treatment on Aβ aggregates in animals with preexisting pathology

β1-antibody treatment of APP23 mice between 7 and 11 months neither led to any improvement of neurodegenerative lesions nor to changes in the levels of soluble or the insoluble types of Aβ aggregates or in Aβ plaque-loads (Fig. 8). Modified forms of Aβ occurred in PBS- and β1-treated APP23 mice in similar amounts (Fig. 8). Interestingly, detection of Aβ plaques in β1-treated mice was attenuated for staining with the β1-antibody and the B10AP antibody fragments recognizing protofibril/ fibril-related epitopes. In contrast, immunoprecipitation of soluble and dispersible fractions with A11 and B10AP revealed similar levels of soluble and dispersible Aβ oligomers, protofibrils and fibrils. It is tempting to speculate that β1 antibody bound to plaques to a certain extent blocked binding of further β1 and B10AP antibodies. This finding is in agreement with the binding of intravenously injected β1 antibody to preexisting plaques in APP23 mice [49].

However, immunoprecipitation of antibody-bound peptides by incubating brain homogenates with protein G-coated beads revealed no significant differences between 11-month-old immunized and non-immunized animals although antibody-bound Aβ was immunoprecipitated in the serum of β1-treated animals in contrast to PBS-treated ones. The unexpected finding of IgG in plaques and bound to Aβ aggregates in PBS-treated and β1-treated animals in similar amounts may be explained by naturally occurring autoantibodies that interact with amyloid plaques in APP23 mice as previously demonstrated in AD patients [57, 58]. Further evidence in favor of this interpretation is provided by our finding that IgG was detected immunohistochemically in plaques of PBS- and β1-treated animals in similar amounts and pattern.

### Aspects of Aβ maturation for Aβ-immunotherapy

The major finding of this study is that the maturation stage of Aβ aggregates has impact on protective effects of Aβ immunotherapy related to Aβ toxicity. If this effect of the maturation stage on Aβ-immunotherapy seen in the APP23 mouse would also apply for human AD this would mean that immunotherapy should be started in early preclinical stages of the disease exhibiting very few plaques and B-Aβ stage 1. The non-favorable outcomes of recently completed trials on passive immunization against Aβ with bapineuzumab or solanezumab [13, 14] could be explained as a logical consequence of the selection of patients with early symptomatic AD, i.e. patients that very likely already had B-Aβ stage 3 [17] indicative for advanced stage Aβ pathology when they started the trial. Accordingly, it still seems reasonable that Aβ-targeting immunotherapy trials which include non-diseased individuals in a preclinical phase of AD instead of symptomatic AD patients with late stage pathology, may show a protective effect as shown here in APP23 mice. However, in our mouse model we only studied the effects of Aβ but not that of τ-pathology, which prevails in human AD brain as well [59, 60] and appears capable of spreading into further brain regions even without being triggered by Aβ [61].

## Conclusions

Our results confirm previous studies that the protective potential of passive Aβ immunization is much greater than its disease modifying potential and demonstrate for the first time that the maturation stage of the biochemical Aβ aggregate composition, i.e. the B-Aβ stage, has impact on Aβ-antibody treatment. As such, our results point to an important role of the B-Aβ stage for design of treatment strategies targeting Aβ.

## Additional files

Additional file 1: Table S1. List of antibodies. Additional file 2: Table S2. Types of commissural neurons traced with DiI [24]. Additional file 3: Table S3. Statistical analysis. Additional file 4: Figure S1. Neuronal and synaptic pathology: Effects of β1 antibody treatment. No differences between PBS- and β1-treated APP23 mice at 5 and 11 months of age in the densities of asymmetric (a) and symmetric synapses in the frontocentral cortex (b) as well as in the numbers of CA1 neurons in the hippocampus (c). Graphs represent mean values (symbols) and standard errors (whiskers). (Statistical analysis in Additional file 3: Table S3). Additional file 5: Figure S2. Neuritic plaques: Effects of β1 antibody treatment. APP-type neuritic plaques (arrows) are detectable in both anti-Aβ (β1)- (a) and PBS-treated 11-month-old APP23 mice (b). Calibration bar in b (valid for a, b) = 27.5 μm. Additional file 6: Figure S3. IgG accumulation in plaques and cerebral amyloid angiopathy and microglial activation: Effects of β1 antibody treatment. Detection of microglial cells and mouse IgG in 5- and 11-month-old β1- and PBS-treated APP23 mice. Mouse IgG comprises intrinsic mouse IgG and the mouse-IgG antibody β1 used for treatment. Amyloid plaques in 11-month-old mice were detected by Aβ autofluorescence [35] whereas no autofluorescent plaques were found in 5 month old animals. a–h: In 5-month-old APP23 mice no IgG and no amyloid material was observed in both β1 and PBS-treated mice. Iba-1 immunostaining showed comparable microglia pattern in both groups. i–t: Mouse IgG occurred in all plaques (white and lucent arrows) and cerebral amyloid angiopathy (arrowheads) affected vessels identified by amyloid autofluorescence in 11-month-old APP23 mice. Plaque-associated microglial activation was observed in β1- and PBS-treated mice in this age group (white arrows). Calibration bar in a (valid for a-t) = 60 μm. Additional file 7: Figure S4. IgG accumulation in plaques and cerebral amyloid angiopathy and astroglial activation: Effects of β1 antibody treatment. Detection of astrocytes and mouse IgG in 5- and 11-month-old β1- and PBS-treated APP23 mice. Mouse IgG comprises intrinsic mouse IgG and the mouse-IgG antibody β1 used for treatment. Amyloid plaques in 11-month-old mice were detected by Aβ autofluorescence [35] whereas no autofluorescent plaques were found in 5 month old animals. a–h: In 5-month-old APP23 mice no IgG and no amyloid material was observed in both β1 and PBS-treated mice. GFAP immunostaining showed comparable astroglia pattern in both groups. i–p: Mouse IgG occurred in all plaques and cerebral amyloid angiopathy affected vessels identified by amyloid autofluorescence in 11-month-old APP23 mice. Plaque-associated astrogliosis was observed in β1- and PBS-treated mice in this age group. Calibration bar in a (valid for a–l) = 80 μm; (m–p) = 70 μm. Additional file 8: Figure S5. Soluble, dispersible, membrane-associated, and plaque-associated Aβ in APP23 mice: Effects of β1 antibody treatment. Full-length images of western blots corresponding to the semiquantitative data shown in Fig. 4: Aβ, Aβ_N3pE_, and pAβ in the soluble, dispersible, membrane-associated, and plaque-associated fraction of brain homogenates of PBS- and β1-treated 5- and 11-month-old APP23 mice. No differences between PBS- and β1-treated animals except for plaque-associated Aβ in 5-month-old mice: β1-treated animals exhibited slightly more non-modified plaque-associated Aβ than non-treated mice. Aβ_N3pE_ and pAβ were not detected in brain homogenates of 5-month-old APP23 mice but in the dispersible, membrane-associated, and plaque-associated fraction of 11-month-old mice without differences in relation to the treatment. The staining pattern of the blots may also exhibit oligomeric Aβ aggregates. Since SDS-PAGE do not provide the native oligomer pattern [37] we focused our data analysis on the monomer band that has been demonstrated to reflect best the Aβ content when performing SDS-PAGE analysis [23]. Additional file 9: Figure S6. Soluble and dispersible (insoluble) Aβ fibrils/protofibrils and non-fibrillar oligomers in APP23 mice: Effects of β1 antibody treatment. Full-length images of western blots corresponding to the semiquantitative data shown in Fig. 5: Western blots from soluble and dispersible non-fibrillar oligomers immunoprecipitated with A11 and protofibrils and fibrils immunoprecipitated with B10AP from the respective brain homogenate fractions in 5- and 11-month-old β1- and PBS-treated APP23 mice. 5-month-old β1-treated mice exhibited soluble Aβ-oligomers, protofibrils and fibrils that were not seen in PBS-treated controls. Although dispersible Aβ oligomers, protofibrils and fibrils were detected in 5-month-old APP23 mice as well there were no differences between β1- and PBS-treated mice. Aβ_N3pE_ and pAβ were not seen in 5-month-old mice. No differences in soluble and dispersible Aβ aggregates were observed between 11-month-old β1- and PBS-treated mice. Aβ_N3pE_ and pAβ were not detectable in soluble oligomers, protofibrils and fibrils as well as in dispersible oligomers at both ages whereas dispersible fibrils and protofibrils exhibited both Aβ_N3pE_ and pAβ. Additional file 10: Figure S7. Immunoglobulin-bound Aβ detected in oligomers, protofibrils and fibrils in APP23 mice: Effects of β1 antibody treatment. Full-length images of western blots corresponding to the semiquantitative data shown in Fig. 6. Western blots after immunoprecipitation of immunoglobulin-bound Aβ by precipitation of antibodies with protein G-coated magnetic beads. Subsequent western blot analysis with anti-Aβ_1–17_ revealed antibody-bound Aβ in the dispersible fraction of both β1- and PBS-treated mice at both ages. In 5-month-old β1-treated APP23 mice antibody-bound Aβ was found in the soluble fraction whereas no antibody-bound Aβ was precipitated in PBS-treated animals. At 11-months of age both, β1 and PBS-treated animals exhibited antibody bound soluble Aβ in similar amounts. Antibody-bound Aβ_N3pE_ was not observed whereas 11-month-old (but not 5-month-old) APP23 mice showed similar amounts of dispersible antibody-bound pAβ. No soluble antibody-bound pAβ was seen. Additional file 11: Figure S8. Serum Aβ immunoprecipitated by protein-G bound antibodies in APP23 mice: Effects of β1 antibody treatment. Full-length images of western blots corresponding to the semiquantitative data shown in Fig. 7. Western blot analysis of blood serum for Aβ and immunoprecipitation of intrinsic serum antibodies by incubation with protein G-coated magnetic beads and with subsequent western blotting for Aβ. Aβ was only seen in 5-month-old β1-treated APP23 mice after antibody-immunoprecipitation and detection with anti-Aβ_1–17_ (6E10). Aβ_N3pE_ and pAβ were not found in these precipitates. PBS-treated and 5-month-old mice did not exhibit detectable amounts of Aβ.
