# Supplementary material for: Impact of amyloid β aggregate maturation on antibody treatment in APP23 mice
Source: Acta Neuropathol Commun. 2015 Jul 4;3:41. doi: 10.1186/s40478-015-0217-z (PMC4491274; doi:10.1186/s40478-015-0217-z)
Supplement: Additional file 1: Table S1. — List of antibodies. [file 40478_2015_217_MOESM1_ESM.doc]

**Additional File 1: Table S1:** Staining methods and antibodies used for detection of amyloid material and neurofibrillary aggregates. (+ = positive; (+) partially positive; - = negative; n.a. = not assessed; IP = immunoprecipitation; WB = western blot)

| **antibody/ staining method** | **amyloid plaques** | **Aβ protofibrils/ fibrils** | **Aβ oligomers** | **neuritic plaques** | **clone/name, distributor, reference** | **dilution, pretreatment for immunohistochemistry** | **dilution for biochemistry** |
| --- | --- | --- | --- | --- | --- | --- | --- |
| **anti-Aβ_17-24_**  detects Aβ_1-40/42_ | + | + | + | + | 4G8, Covance, Dedham, USA | 1/5,000, formic acid pretreatment | n.a. |
| **anti-Aβ_1-17_**  detects Aβ_1-40/42_  no crossreaction with pAβ [18] | + | + | + | + | 6E10, Covance, Dedham USA, | 1/1000, formic acid pretreatment | IP: 1µl/200µl lysate  WB: 1/1000 |
| **anti-Aβ_42_**  detects Aβ_1-42_ | + | + | + | + | MBC42,[31] | 1/200; formic acid pretreatment | n.a. |
| **anti-Aβ_40_**  detects Aβ_1-40_ | + | + | + | + | polycloncal anti-Aβ_40_, IBL | 1/100, formic acid pretreatment | n.a. |
| **β1**  detects human N-terminal of Aβ,  the epitope is between Aβ_3-6_ | + | + | + | + | Β1, [28,29] | 0,46 µg/ml, formic acid pretreatment | n.a. |
| **anti-oligomer (A11)**  detects oligomers of Aβ and  other proteins | **-** | - | + | - | A11, Millipore, Temecula,CA, USA, [62] | 1/1000, microwave pretreatment | IP: 1µl/200µl lysate |
| **anti-protofibrillar/fibrillar, B10AP-antibody fragments**  detects protofibrils and fibrils  of Aβ and other proteins | (+) | + | - | (+) | B10AP, [34] | 1/10, microwave pretreatment | IP: 1µl/200µl lysate |
| **anti-pAβ**  detects pAβ phosphorylated  at serine 8 | (+) | (+) | (+) | (+) | IE4E11, [63] | 1/50, formic acid and microwave pretreatment | WB: 1/100 |
| **anti-Aβ_N3pE_**  detects Aβ_N3pE_ | (+) | (+) | (+) | (+) | Polyclonal rabbit,IBL, [19] | 1/100; formic acid and microwave pretreatment | WB: 1/500 |
| **anti-APP**  detects APP and APP-N-terminal fragments | (+) | - | - | + | 22C11, Millipore, Temecula, CA, USA | 1/75, microwave pretreatment | n.a. |
| **anti-MS-IgG**  detects mouse IgG | (+) | (+) | (+) | (+) | Biotin-labeled goat anti-MS-IgG,  Vector laboratories Burlingame, CA, USA | 1/200 | n.a. |
| **Anti-glial fibrillary acidic protein (GFAP)**  detects astrocytes | - | - | - | + | Polyclonal rabbit, DAKO, Glostrup, Denmark | 1/1000 | n.a. |
| **Anti-iba-1**  detects micrglial cells | - | - | - | + | Polyclonal rabbit, Wako, Richmond, VA, USA | 1/500 | n.a. |
