# Supplementary material for: Impact of amyloid β aggregate maturation on antibody treatment in APP23 mice
Source: Acta Neuropathol Commun. 2015 Jul 4;3:41. doi: 10.1186/s40478-015-0217-z (PMC4491274; doi:10.1186/s40478-015-0217-z)
Supplement: Additional file 2: Table S2. — Types of commissural neurons traced with DiI [24]. [file 40478_2015_217_MOESM2_ESM.doc]

**Additional File 2: Table S2**: Types of commissural neurons in layer III of the frontocentral cortex identified by DiI-tracing [24].

| Type I commissural neurons | Pyramidal neurons with a highly ramified dendritic tree showing multiple secondary and tertiary branches |
| --- | --- |
| Type II commissural neurons | Pyramidal neurons with a dendritic tree that branches distant from the cell soma, secondary and tertiary branches are usually not observed in 100µm thick sections |
| Type III commissural neurons | Non-pyramidal commissural neurons |
