# Supplementary material for: Impact of amyloid β aggregate maturation on antibody treatment in APP23 mice
Source: Acta Neuropathol Commun. 2015 Jul 4;3:41. doi: 10.1186/s40478-015-0217-z (PMC4491274; doi:10.1186/s40478-015-0217-z)
Supplement: Additional file 3: Table S3. — Statistical analysis. [file 40478_2015_217_MOESM3_ESM.doc]

**Additional file 3: Table S3**. Statistical analysis

a. *DiI tracing of commissural neurons in the frontocentral cortex*

*DiI-tracing of commissural neurons: Mann-Whitney U-test (exact test – single sided)*

*Comparison between β1- and PBS-treated mice*

| ***Treatment between:*** | **Type I neurons** | **Type II neurons** | **Type III neurons** |
| --- | --- | --- | --- |
| 3-5 months | p = 0.027  β1 > PBS | p = 0.419 | p = 0.459 |
| 7-11 months | p = 0.456 | p = 0.485 | p = 0.218 |

n = 10 (each group), except β1-treated mice at 5 months of age: n = 7; and PBS-treated mice at 5 months of age: n = 9

*b. Number of hippocampal synapses in stratum oriens and radiatum of CA1: Mann-Whitney U-test (exact test – two sided)*

*Comparison between β1- and PBS-treated mice*

| ***Treatment between:*** | ***Asymmetric synapses*** | ***Symmetric synapses*** |
| --- | --- | --- |
| 3-5 months | p = 0.589 | p = 1 |
| 7-11 months | p = 0.31 | p = 0.485 |

n = 6 (each group)

*c. Number of hippocampal CA1 neurons*

*β1-treated vs. PBS-treated: Welch-test*

| ***Treatment between:*** | **p** |
| --- | --- |
| 3-5 months | 0.453 |
| 7-11 months | 0.098 |

n = 6 (each group)

*d. Plaque loads: Mann-Whitney U-test (exact test – two sided)*

*Comparison between β1- and PBS-treated mice*

| **Treatment between:** | **Aβ_42_** | **Aβ_40_** | **Aβ_N3pE_** | **pAβ** | **β1** | **B10AP** |
| --- | --- | --- | --- | --- | --- | --- |
| 3-5 months | p = 0.243 | p = 0.699 | p = 0.699 | p = 0.699 | p = 1 | p = 1 |
| 7-11 months | p = 0.853 | p = 0.699 | p = 0.699 | p = 0.426 | p = 0.045  β1 < PBS | p = 0.015  β1 < PBS |

n = 6 (each group); except for Aβ_42_ (both age groups), β1 and B10AP (7-11 months): n = 10.

*e. Soluble, dispersible, membrane-associated, and plaque-associated Aβ, Aβ_N3pE_, and pAβ*

*Comparison between β1- and PBS-treated mice by Student-t-test/ Welch-test*

n indicates the numbers of animals in each treatment group.

| **Treatment between:** | **Soluble Aβ** | **Dispersible Aβ** | **Membrane-associated Aβ** | **Plaque-associated Aβ** |
| --- | --- | --- | --- | --- |
| 3-5 months | p = 0.632 | p = 0.148 | p = 0.260 | p = 0.002  β1 > PBS |
| 7-11 months | p = 0.153 | p = 0.297 | p = 0.397 | p = 0.217 |

Soluble, dispersible Aβ: n = 5; membrane-associated, plaque-associated Aβ: n = 6

| **Treatment between:** | **Soluble Aβ_N3pE_** | **Dispersible Aβ_N3pE_** | **Membrane-associated Aβ_N3pE_** | **Plaque-associated Aβ_N3pE_** |
| --- | --- | --- | --- | --- |
| 3-5 months | p = 0.151 | p = 0.095 | p = 0.260 | p = 0.081 |
| 7-11 months | p = 0.272 | p = 0.326 | p = 0.620 | p = 0.053 |

Soluble, dispersible Aβ: n = 5; membrane-associated, plaque-associated Aβ: n = 6

| **Treatment between:** | **Soluble pAβ** | **Dispersible pAβ** | **Membrane-associated pAβ** | **Plaque-associated pAβ** |
| --- | --- | --- | --- | --- |
| 3-5 months | p = 0.695 | p = 0.608 | p = 0.996 | p = 0.367 |
| 7-11 months | p = 0.070 | p = 0.070 | p = 0.302 | p = 0.358 |

Soluble, dispersible Aβ: n = 5; membrane-associated: n = 6, plaque-associated Aβ: n = 3 (5 months), 6 (11 months)

*f. Soluble, dispersible oligomers (IP with A11), protofibrils and fibrils (IP with B10AP) containing Aβ, Aβ_N3pE_, and pAβ*

*Comparison between β1- and PBS-treated mice by Student-t-test/ Welch-test*

n indicates the numbers of animals in each treatment group.

|  | **IP-A11: nonfibrillar oligomers** | | **IP-B10AP: protofibrils and fibrils** | |
| --- | --- | --- | --- | --- |
| **Treatment between:** | **Soluble Aβ** | **Dispersible Aβ** | **Soluble Aβ** | **Dispersible Aβ** |
| 3-5 months | p = 0.030  β1 > PBS | p = 0.695 | p = 0.043  β1 > PBS | p = 0.216 |
| 7-11 months | p = 0.513 | p = 0.270 | p = 0.234 | p = 0.410 |

n = 3

|  | **IP-A11: nonfibrillar oligomers** | | **IP-B10AP: protofibrils and fibrils** | |
| --- | --- | --- | --- | --- |
| **Treatment between:** | **Soluble Aβ_N3pE_** | **Dispersible Aβ_N3pE_** | **Soluble Aβ_N3pE_** | **Dispersible Aβ_N3pE_** |
| 3-5 months | p = 0.385 | p = 0.136 | p = 0.970 | p = 0.920 |
| 7-11 months | p = 0.363 | p = 0.981 | p = 0.530 | p = 0.124 |

n = 3

|  | **IP-A11: nonfibrillar oligomers** | | **IP-B10AP: protofibrils and fibrils** | |
| --- | --- | --- | --- | --- |
| **Treatment between:** | **Soluble pAβ** | **Dispersible pAβ** | **Soluble pAβ** | **Dispersible pAβ** |
| 3-5 months | p = 0.132 | p = 0.421 | p = 0.750 | p = 0.663 |
| 7-11 months | p = 0.162 | p = 0.460 | p = 0.273 | p = 0.099 |

n = 3

*g. Soluble and dispersible antibody-bound Aβ, Aβ_N3pE_, and pAβ precipitated with protein G-coated magnetic beads.*

*Comparison between β1- and PBS-treated mice by Student-t-test/ Welch-test*

n indicates the numbers of animals in each treatment group.

|  | **IP-intrinsic IgG in brain** | |
| --- | --- | --- |
| **Treatment between:** | **Soluble Aβ** | **Dispersible Aβ** |
| 3-5 months | p = 0.002  β1 > PBS | p = 0.134 |
| 7-11 months | p = 0.059 | p = 0.433 |

n = 3

|  | **IP-intrinsic IgG in brain** | |
| --- | --- | --- |
| **Treatment between:** | **Soluble Aβ_N3pE_** | **Dispersible Aβ_N3pE_** |
| 3-5 months | p = 0.461 | p = 0.186 |
| 7-11 months | p = 0.740 | p = 0.880 |

n = 3

|  | **IP-intrinsic IgG in brain** | |
| --- | --- | --- |
| **Treatment between:** | **Soluble pAβ** | **Dispersible pAβ** |
| 3-5 months | p = 0.802 | p = 0.210 |
| 7-11 months | p = 0.952 | p = 0.094 |

n = 3

*h. Antibody-bound Aβ, Aβ_N3pE_, and pAβ in the blood serum precipitated with protein G-coated magnetic beads.*

*Comparison between β1- and PBS-treated mice by Student-t-test/ Welch-test*

n indicates the numbers of animals in each treatment group.

|  | **IP-intrinsic IgG in serum** | | |
| --- | --- | --- | --- |
| **Treatment between:** | **Aβ** | **Aβ_N3pE_** | **pAβ** |
| 3-5 months | p = 0.215 | p = 0.342 | p = 0.262 |
| 7-11 months | p = 0.017  β1 > PBS | p = 0.231 | p = 0.368 |

n = 5, Aβ_N3pE_: n = 6
