# Supplementary figures and images for: Impact of amyloid β aggregate maturation on antibody treatment in APP23 mice
Source: Acta Neuropathol Commun. 2015 Jul 4;3:41. doi: 10.1186/s40478-015-0217-z (PMC4491274; doi:10.1186/s40478-015-0217-z)

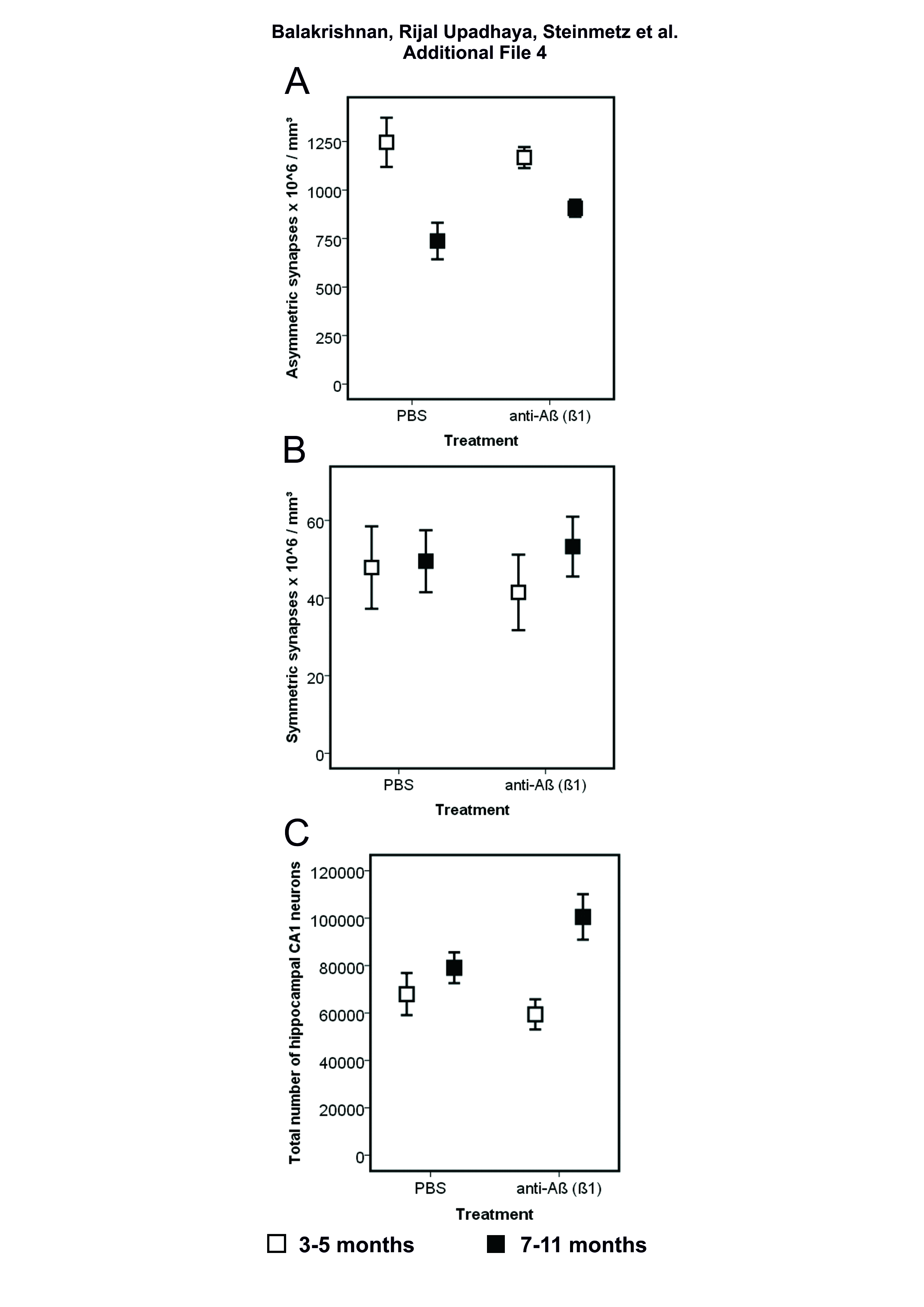

Supplement: Additional file 4: Figure S1. — Neuronal and synaptic pathology: Effects of β1 antibody treatment. No differences between PBS- and β1-treated APP23 mice at 5 and 11 months of age in the densities of asymmetric (a) and symmetric synapses in the frontocentral cortex (b) as well as in the numbers of CA1 neurons in the hippocampus (c). Graphs represent mean values (symbols) and standard errors (whiskers). (Statistical analysis in Additional file 3: Table S3). [file 40478_2015_217_MOESM4_ESM.tif]

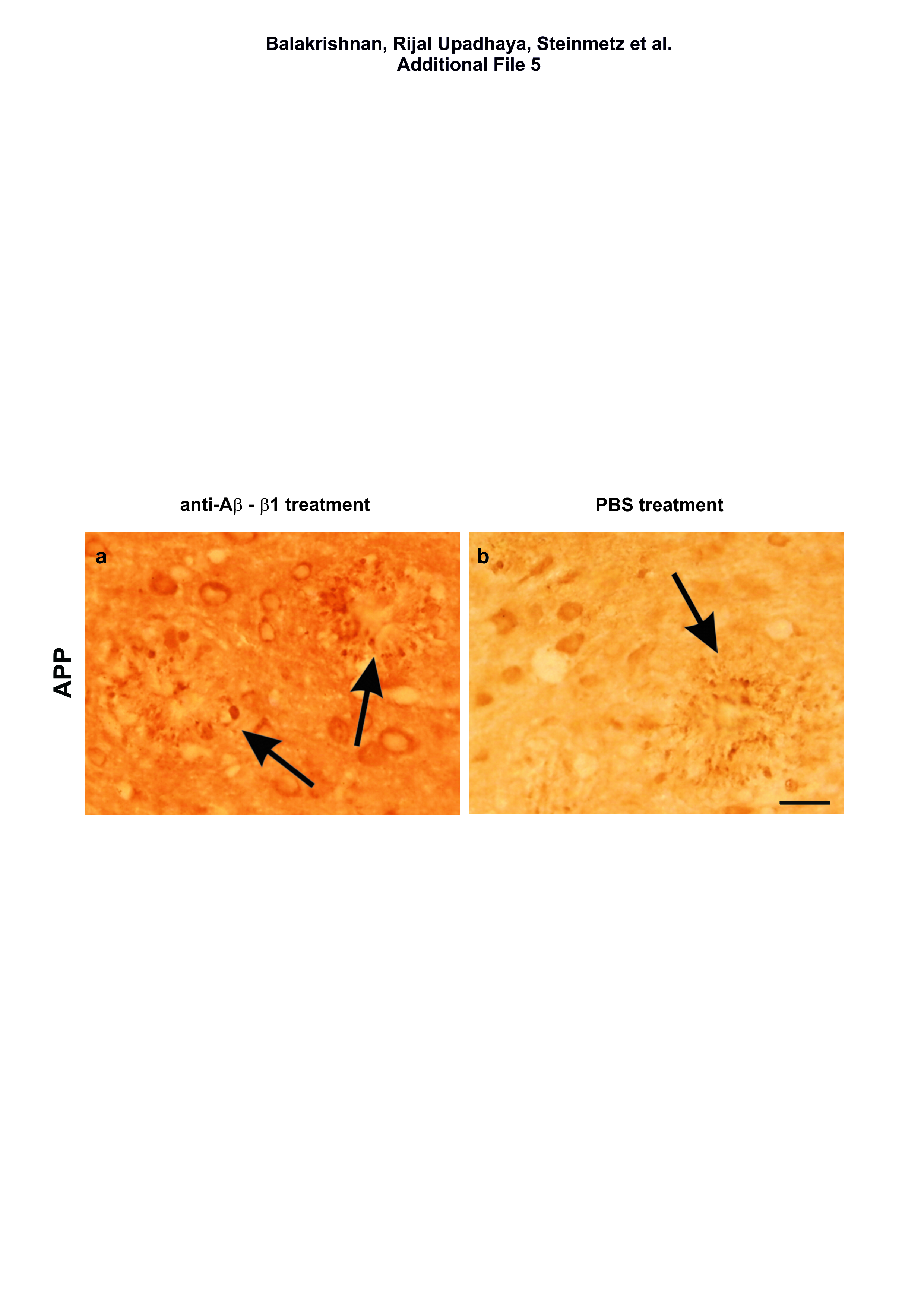

Supplement: Additional file 5: Figure S2. — Neuritic plaques: Effects of β1 antibody treatment. APP-type neuritic plaques (arrows) are detectable in both anti-Aβ (β1)- (a) and PBS-treated 11-month-old APP23 mice (b). Calibration bar in b (valid for a, b) = 27.5 μm. [file 40478_2015_217_MOESM5_ESM.tif]

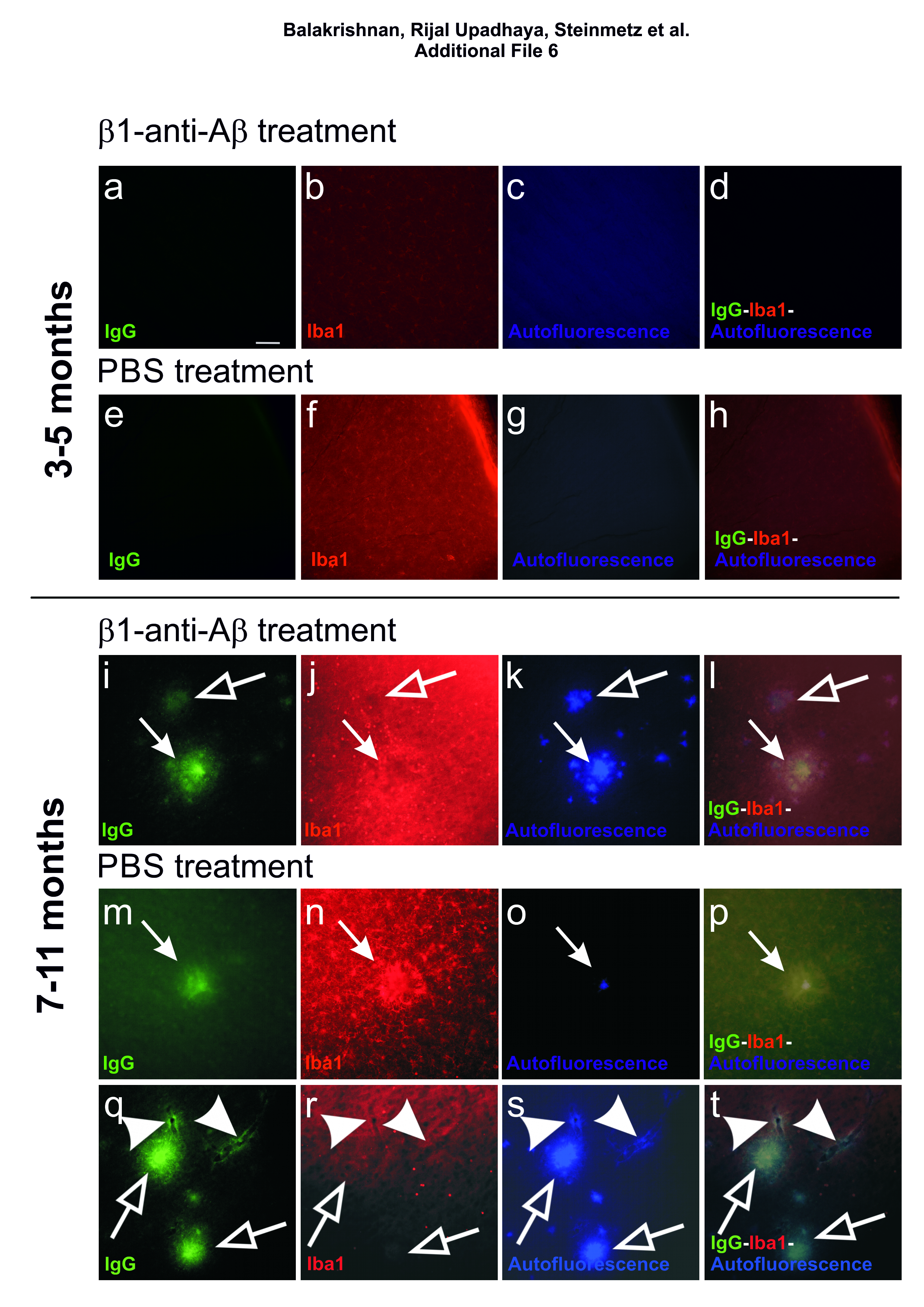

Supplement: Additional file 6: Figure S3. — IgG accumulation in plaques and cerebral amyloid angiopathy and microglial activation: Effects of β1 antibody treatment. Detection of microglial cells and mouse IgG in 5- and 11-month-old β1- and PBS-treated APP23 mice. Mouse IgG comprises intrinsic mouse IgG and the mouse-IgG antibody β1 used for treatment. Amyloid plaques in 11-month-old mice were detected by Aβ autofluorescence [35] whereas no autofluorescent plaques were found in 5 month old animals. a–h: In 5-month-old APP23 mice no IgG and no amyloid material was observed in both β1 and PBS-treated mice. Iba-1 immunostaining showed comparable microglia pattern in both groups. i–t: Mouse IgG occurred in all plaques (white and lucent arrows) and cerebral amyloid angiopathy (arrowheads) affected vessels identified by amyloid autofluorescence in 11-month-old APP23 mice. Plaque-associated microglial activation was observed in β1- and PBS-treated mice in this age group (white arrows). Calibration bar in a (valid for a-t) = 60 μm. [file 40478_2015_217_MOESM6_ESM.tif]

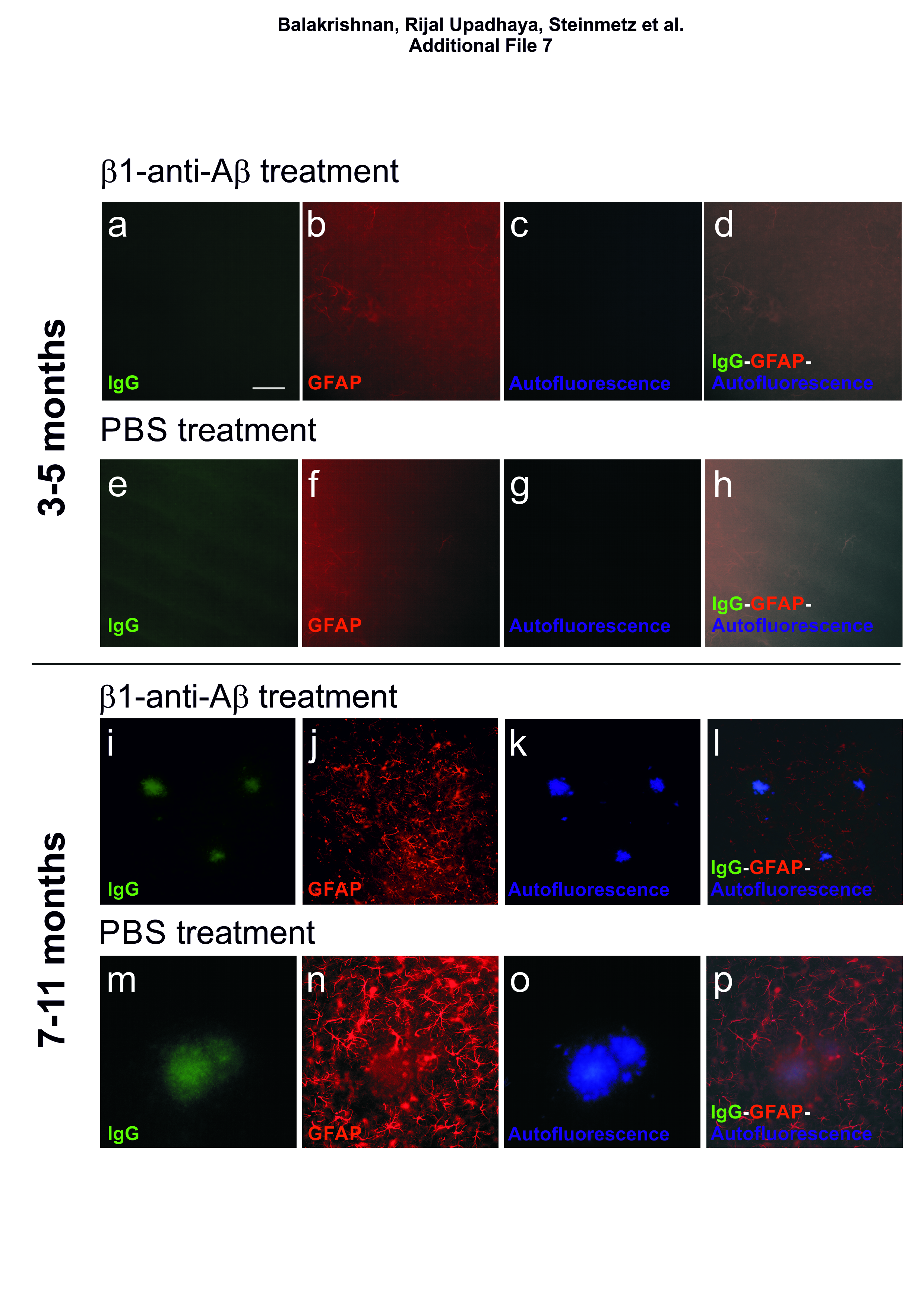

Supplement: Additional file 7: Figure S4. — IgG accumulation in plaques and cerebral amyloid angiopathy and astroglial activation: Effects of β1 antibody treatment. Detection of astrocytes and mouse IgG in 5- and 11-month-old β1- and PBS-treated APP23 mice. Mouse IgG comprises intrinsic mouse IgG and the mouse-IgG antibody β1 used for treatment. Amyloid plaques in 11-month-old mice were detected by Aβ autofluorescence [35] whereas no autofluorescent plaques were found in 5 month old animals. a–h: In 5-month-old APP23 mice no IgG and no amyloid material was observed in both β1 and PBS-treated mice. GFAP immunostaining showed comparable astroglia pattern in both groups. i–p: Mouse IgG occurred in all plaques and cerebral amyloid angiopathy affected vessels identified by amyloid autofluorescence in 11-month-old APP23 mice. Plaque-associated astrogliosis was observed in β1- and PBS-treated mice in this age group. Calibration bar in a (valid for a–l) = 80 μm; (m–p) = 70 μm. [file 40478_2015_217_MOESM7_ESM.tif]

Balakrishnan, Rijal Upadhaya, Steinmetz et al.  
Additional File 8

**a**

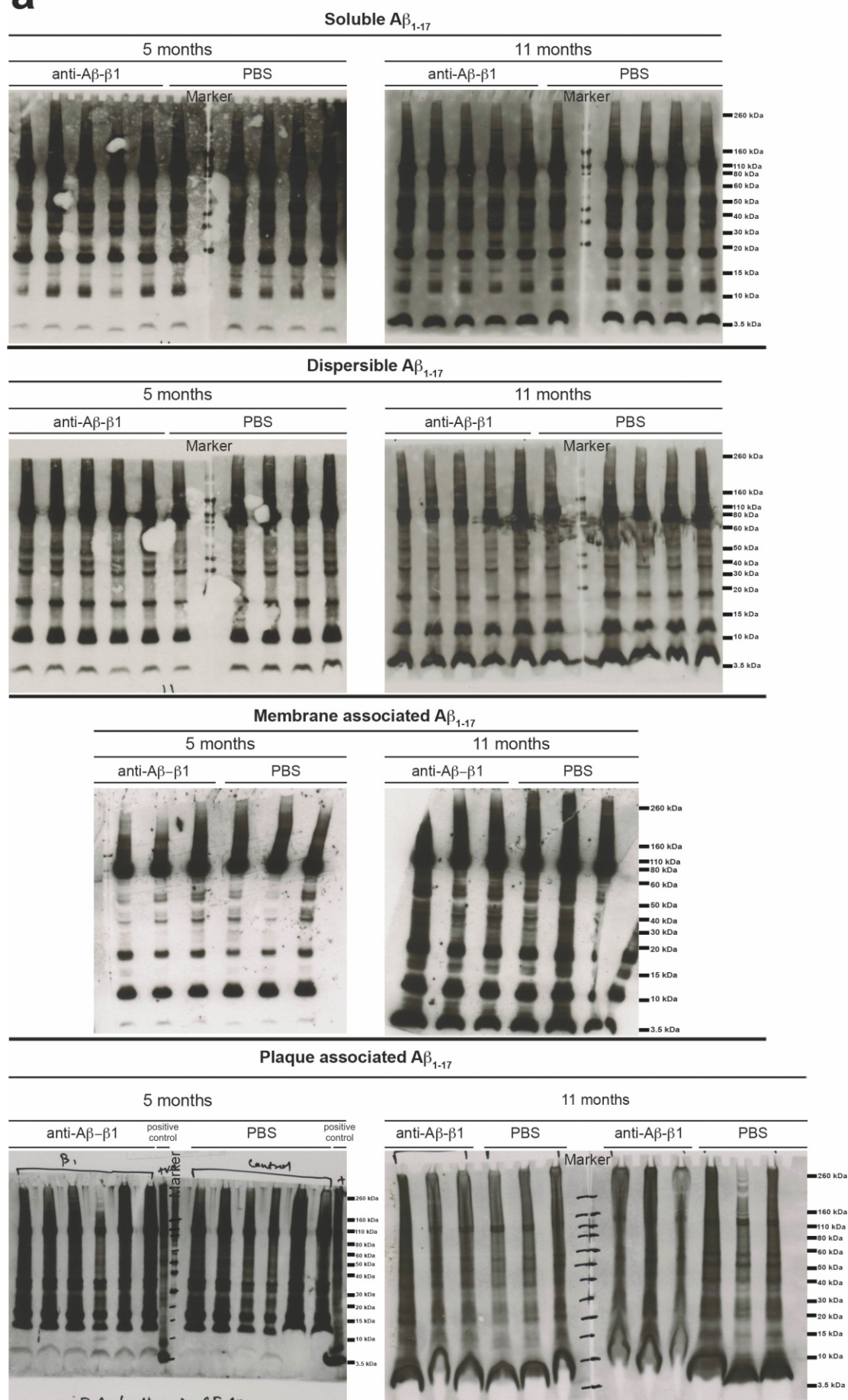

b

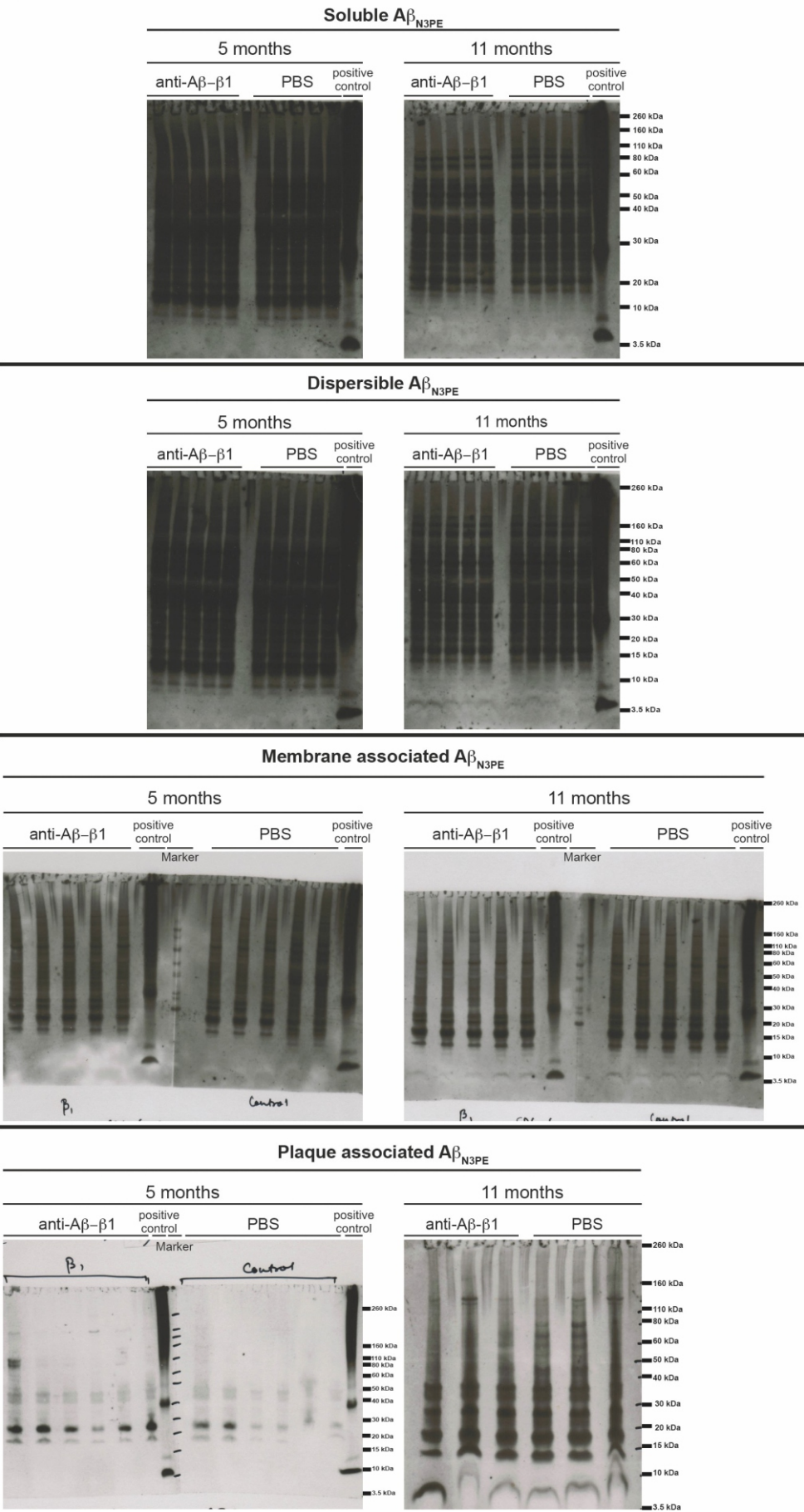

**C**

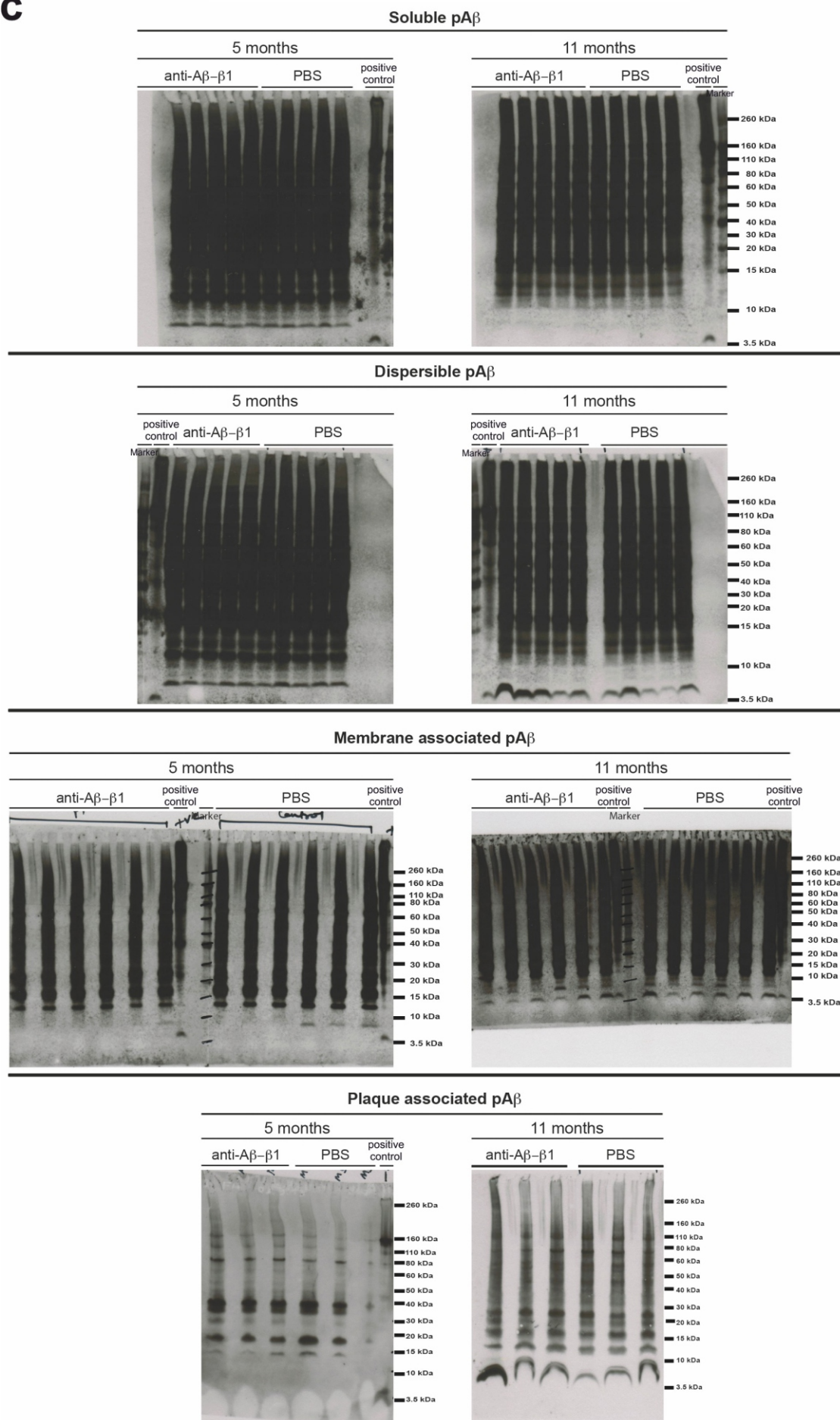

Supplement: Additional file 8: Figure S5. — Soluble, dispersible, membrane-associated, and plaque-associated Aβ in APP23 mice: Effects of β1 antibody treatment. Full-length images of western blots corresponding to the semiquantitative data shown in Fig. 4: Aβ, AβN3pE, and pAβ in the soluble, dispersible, membrane-associated, and plaque-associated fraction of brain homogenates of PBS- and β1-treated 5- and 11-month-old APP23 mice. No differences between PBS- and β1-treated animals except for plaque-associated Aβ in 5-month-old mice: β1-treated animals exhibited slightly more non-modified plaque-associated Aβ than non-treated mice. AβN3pE and pAβ were not detected in brain homogenates of 5-month-old APP23 mice but in the dispersible, membrane-associated, and plaque-associated fraction of 11-month-old mice without differences in relation to the treatment. The staining pattern of the blots may also exhibit oligomeric Aβ aggregates. Since SDS-PAGE do not provide the native oligomer pattern [37] we focused our data analysis on the monomer band that has been demonstrated to reflect best the Aβ content when performing SDS-PAGE analysis [23]. [file 40478_2015_217_MOESM8_ESM.pdf]

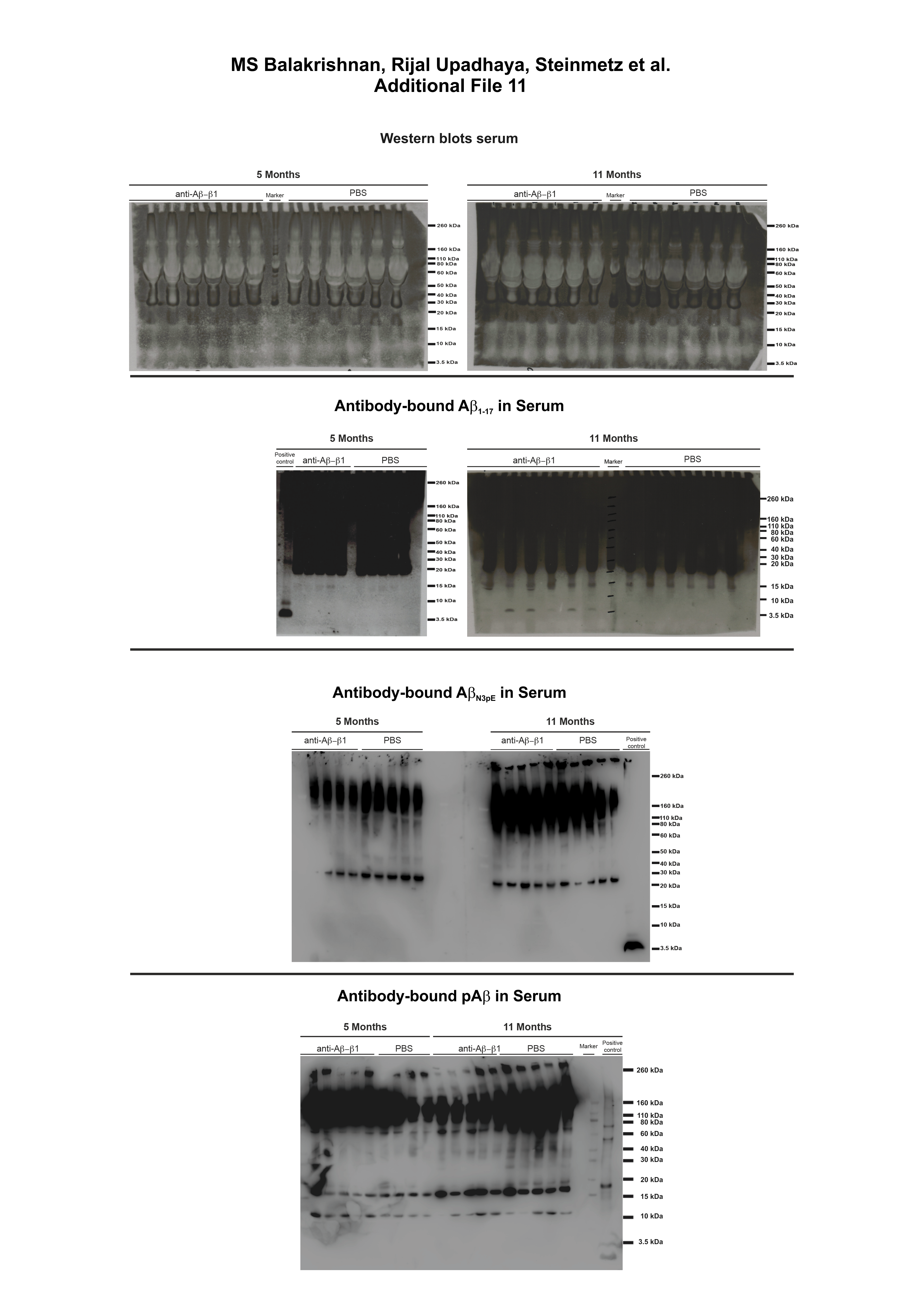

Supplement: Additional file 11: Figure S8. — Serum Aβ immunoprecipitated by protein-G bound antibodies in APP23 mice: Effects of β1 antibody treatment. Full-length images of western blots corresponding to the semiquantitative data shown in Fig. 7. Western blot analysis of blood serum for Aβ and immunoprecipitation of intrinsic serum antibodies by incubation with protein G-coated magnetic beads and with subsequent western blotting for Aβ. Aβ was only seen in 5-month-old β1-treated APP23 mice after antibody-immunoprecipitation and detection with anti-Aβ1–17 (6E10). AβN3pE and pAβ were not found in these precipitates. PBS-treated and 5-month-old mice did not exhibit detectable amounts of Aβ. [file 40478_2015_217_MOESM11_ESM.tif]
