# Supplementary material for: Impact of amyloid β aggregate maturation on antibody treatment in APP23 mice
Source: Acta Neuropathol Commun. 2015 Jul 4;3:41. doi: 10.1186/s40478-015-0217-z (PMC4491274; doi:10.1186/s40478-015-0217-z)

Balakrishnan, Rijal Upadhaya, Steinmetz et al.
Additional File 9

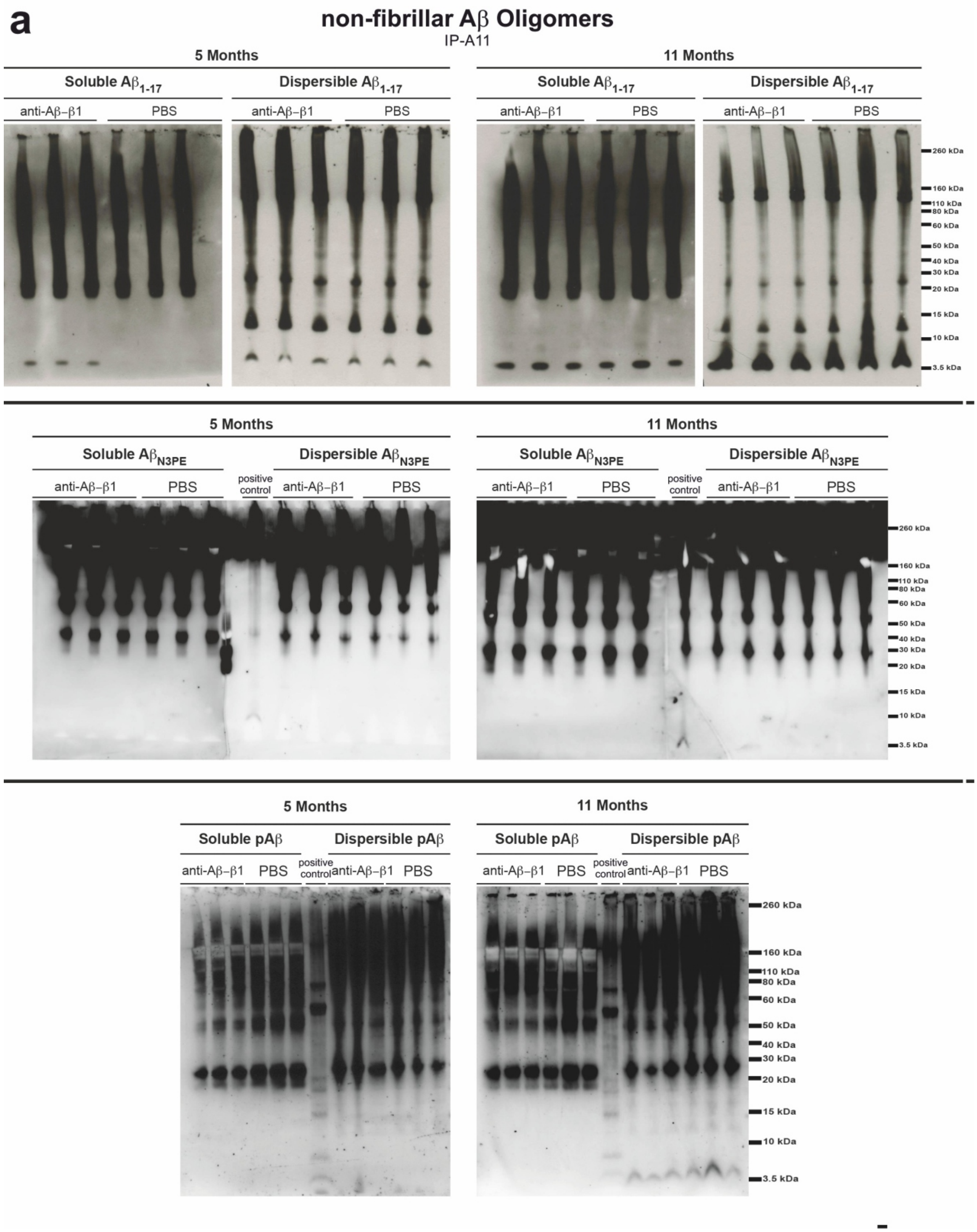

Balakrishnan, Rijal Upadhaya, Steinmetz et al.
Additional File 9

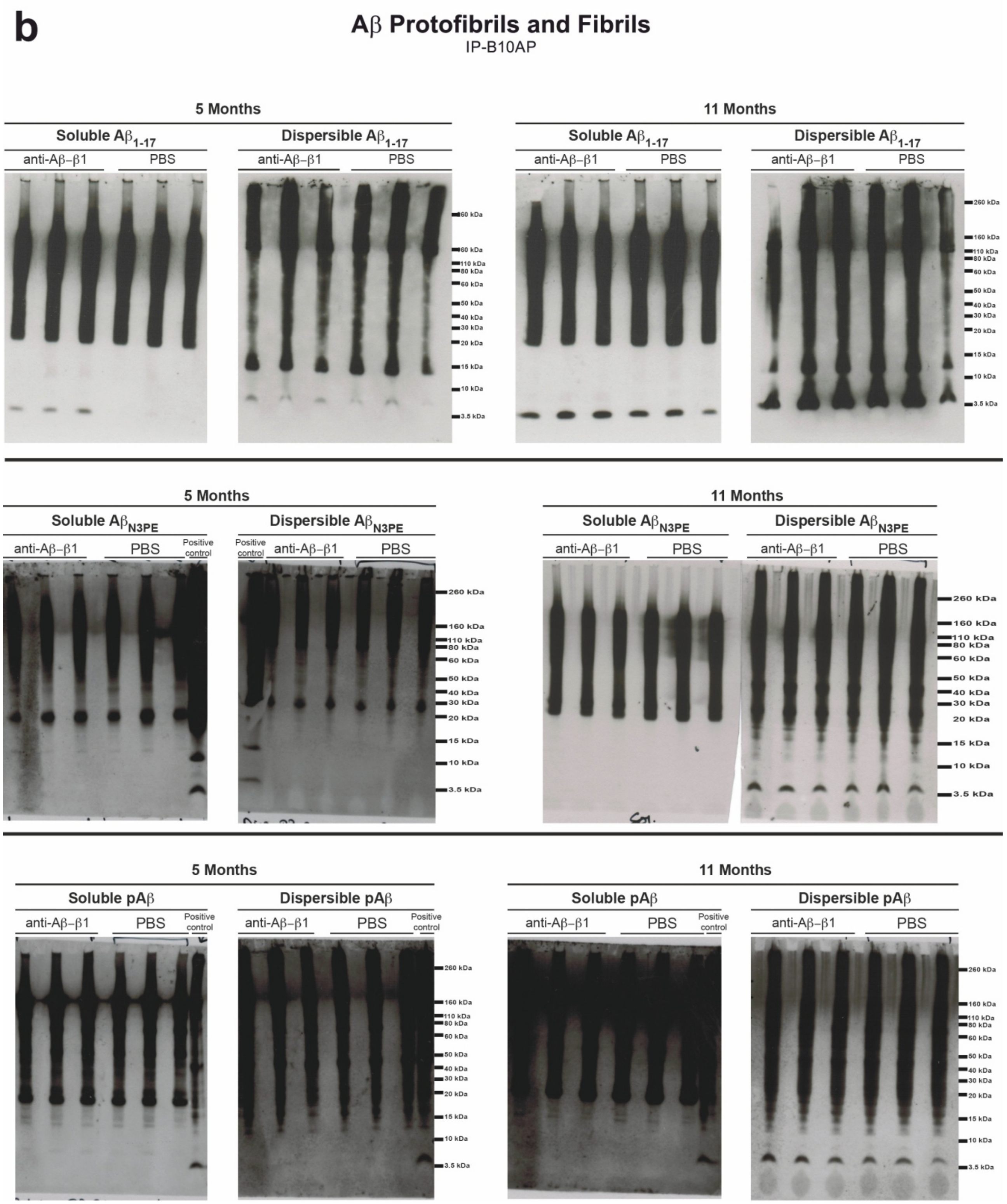

Supplement: Additional file 9: Figure S6. — Soluble and dispersible (insoluble) Aβ fibrils/protofibrils and non-fibrillar oligomers in APP23 mice: Effects of β1 antibody treatment. Full-length images of western blots corresponding to the semiquantitative data shown in Fig. 5: Western blots from soluble and dispersible non-fibrillar oligomers immunoprecipitated with A11 and protofibrils and fibrils immunoprecipitated with B10AP from the respective brain homogenate fractions in 5- and 11-month-old β1- and PBS-treated APP23 mice. 5-month-old β1-treated mice exhibited soluble Aβ-oligomers, protofibrils and fibrils that were not seen in PBS-treated controls. Although dispersible Aβ oligomers, protofibrils and fibrils were detected in 5-month-old APP23 mice as well there were no differences between β1- and PBS-treated mice. AβN3pE and pAβ were not seen in 5-month-old mice. No differences in soluble and dispersible Aβ aggregates were observed between 11-month-old β1- and PBS-treated mice. AβN3pE and pAβ were not detectable in soluble oligomers, protofibrils and fibrils as well as in dispersible oligomers at both ages whereas dispersible fibrils and protofibrils exhibited both AβN3pE and pAβ. [file 40478_2015_217_MOESM9_ESM.pdf]
