# Supplementary material for: Impact of amyloid β aggregate maturation on antibody treatment in APP23 mice
Source: Acta Neuropathol Commun. 2015 Jul 4;3:41. doi: 10.1186/s40478-015-0217-z (PMC4491274; doi:10.1186/s40478-015-0217-z)

Balakrishnan, Rijal Upadhaya, Steinmetz et al.
Additional File 10

Aβ detected in immunoglobulin-containing oligomers, protofibrils and fibrils

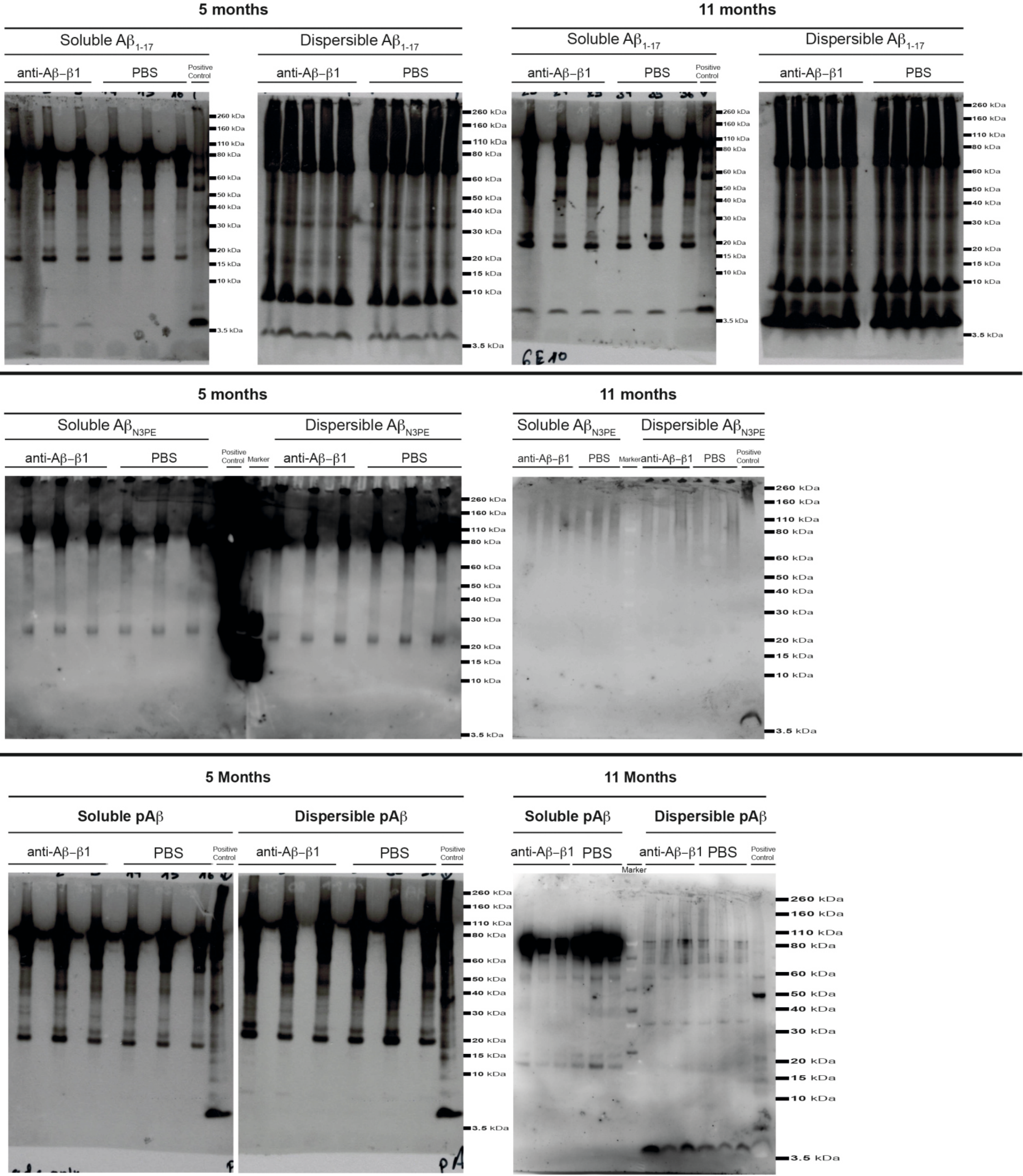

Supplement: Additional file 10: Figure S7. — Immunoglobulin-bound Aβ detected in oligomers, protofibrils and fibrils in APP23 mice: Effects of β1 antibody treatment. Full-length images of western blots corresponding to the semiquantitative data shown in Fig. 6. Western blots after immunoprecipitation of immunoglobulin-bound Aβ by precipitation of antibodies with protein G-coated magnetic beads. Subsequent western blot analysis with anti-Aβ1–17 revealed antibody-bound Aβ in the dispersible fraction of both β1- and PBS-treated mice at both ages. In 5-month-old β1-treated APP23 mice antibody-bound Aβ was found in the soluble fraction whereas no antibody-bound Aβ was precipitated in PBS-treated animals. At 11-months of age both, β1 and PBS-treated animals exhibited antibody bound soluble Aβ in similar amounts. Antibody-bound AβN3pE was not observed whereas 11-month-old (but not 5-month-old) APP23 mice showed similar amounts of dispersible antibody-bound pAβ. No soluble antibody-bound pAβ was seen. [file 40478_2015_217_MOESM10_ESM.pdf]
